# Intragenomic rearrangements involving 5′-untranslated region segments in SARS-CoV-2, other betacoronaviruses, and alphacoronaviruses

**DOI:** 10.1186/s12985-023-01998-0

**Published:** 2023-02-25

**Authors:** Roberto Patarca, William A. Haseltine

**Affiliations:** ACCESS Health International, 384 West Lane, Ridgefield, CT 06877 USA

**Keywords:** Alphacoronaviruses, Betacoronaviruses, Variants, Intragenomic rearrangements

## Abstract

**Background:**

Variation of the betacoronavirus SARS-CoV-2 has been the bane of COVID-19 control. Documented variation includes point mutations, deletions, insertions, and recombination among closely or distantly related coronaviruses. Here, we describe yet another aspect of genome variation by beta- and alphacoronaviruses that was first documented in an infectious isolate of the betacoronavirus SARS-CoV-2, obtained from 3 patients in Hong Kong that had a 5′-untranslated region segment at the end of the *ORF6* gene that in its new location translated into an ORF6 protein with a predicted modified carboxyl terminus. While comparing the amino acid sequences of translated ORF8 genes in the GenBank database, we found a subsegment of the same 5′-UTR-derived amino acid sequence modifying the distal end of ORF8 of an isolate from the United States and decided to carry out a systematic search.

**Methods:**

Using the nucleotide and in the case of SARS-CoV-2 also the translated amino acid sequence in three reading frames of the genomic termini of coronaviruses as query sequences, we searched for 5′-UTR sequences in regions other than the 5′-UTR in SARS-CoV-2 and reference strains of alpha-, beta-, gamma-, and delta-coronaviruses.

**Results:**

We here report numerous genomic insertions of 5′-untranslated region sequences into coding regions of SARS-CoV-2, other betacoronaviruses, and alphacoronaviruses, but not delta- or gammacoronaviruses. To our knowledge this is the first systematic description of such insertions. In many cases, these insertions would change viral protein sequences and further foster genomic flexibility and viral adaptability through insertion of transcription regulatory sequences in novel positions within the genome. Among human *Embecorivus* betacoronaviruses, for instance, from 65% to all of the surveyed sequences in publicly available databases contain inserted 5′-UTR sequences.

**Conclusion:**

The intragenomic rearrangements involving 5′-untranslated region sequences described here, which in several cases affect highly conserved genes with a low propensity for recombination, may underlie the generation of variants homotypic with those of concern or interest and with potentially differing pathogenic profiles. Intragenomic rearrangements thus add to our appreciation of how variants of SARS-CoV-2 and other beta- and alphacoronaviruses may arise.

**Supplementary Information:**

The online version contains supplementary material available at 10.1186/s12985-023-01998-0.

## Background

Coronaviruses (CoVs) are positive, singe stranded RNA viruses of the order *Nidovirales*, family *Coronaviridae*, subfamily *Orthocoronavirinae*, with four genera, namely alpha [α], beta [β], gamma [γ] and delta [δ], which have been further subdivided into 25 subgenera, including five for β-CoVs: *Sarbecovirus*, *Merbecovirus*, *Embecovirus*, *Nobecovirus* and *Hibecovirus* [1], and fifteen for α-CoVs: *Luchacovirus*, *Decacovirus*, *Nyctacovirus*, *Minunacovirus*, *Pedacovirus*, *Colacovirus*, *Myotacovirus*, *Duvinacovirus*, *Setracovirus*, *Rhinacovirus*, *Tegacovirus*, *Minacovirus*, *Sunacovirus*, *Soracovirus*, and *Amalacovirus* [2]. Seven CoVs infect humans; two of the α-genus (the *Duvinacovirus* hCoVs 229E and the *Setracovirus* NL63) and five of the β-genus: the *Sarbecoviruses* severe acute respiratory syndrome (SARS)-CoVs 1 and 2, the latter responsible for a pandemic since 2019 [3–6]; the *Merbecovirus* Middle East respiratory syndrome (MERS) CoV; and the *Embecoviruses* hCoV-OC43 and -HKU1. Human CoVs have a zoonotic origin, with bats as key reservoir [7] and possibly other hosts [8, 9]. Bat β-CoVs related to human CoVs belong to the *Sarbecovirus*, *Nobecovirus*, and *Hibecovirus* subgenera [10–12].

Coronaviruses display substantial genomic plasticity and resilience [13, 14] via recombination, point mutations, deletions, and insertions, which are reported to drive variant emergence, host range, gene expression, transmissibility, immune escape, and virulence [15–20]. The use of an RNA-dependent-RNA polymerase (RdRp)-driven template switching mechanism for transcription and control of structural and accessory gene expression in CoVs [20] has been reported to account for the high frequency of recombination [13, 18, 21–27].

In template switching, a leader transcription regulatory sequence (TRS-L; ACGAAC core in β-CoVs) [28] in the 5′-untranslated region (UTR) interacts with homologous TRS-body (B) elements upstream of viral genes in the last third of the genome (illustrated for SARS-CoV-2 in Additional file 1: Fig. S1A) [29, 30]. Template switching renders the neighborhood of TRS-Bs, especially that for the spike gene, a recombination hotspot during viral transcription [3, 16, 21, 22, 24, 27, 31–34].

Viral subgenomic messenger RNAs contain a 5′-leader sequence that spans from the terminal 5′-cap (m^7^G) structure to the TRS-L and harbors three conserved stem-loop (SL1-3) regulatory elements of gene expression and replication (Additional file 1: Fig. S1B) [35–37]. The TRS-L core sequence and the secondary structure of the leader sequence are conserved within but not among coronavirus genera (Rfam database: http://rfam.xfam.org/covid-19).

The entire 5′-leader nucleotide sequence of SARS-CoV-2, and beyond up to almost SL5 can be translated into a peptide sequence (Additional file 1: Fig. S1B), and although there is no evidence for the functionality of any open reading frame within the UTRs [36, 38], the 5′-leader sequence could be translated after most of it (nucleotides 8–80, including SL1-3 and TRS-L) is duplicated and translocated to the distal end of the accessory ORF6 gene of a SARS-CoV-2 variant with deleted ORFs 7a, 7b and 8 isolated from 3 patients in Hong Kong [39]. We also found that a shorter portion of the 5′-leader sequence (nucleotides 50–75) is duplicated and translocated to the end of the accessory ORF8 gene of a USA variant (accession number: QUP34336) that could be translated into a modified ORF-8 protein, which prompted us to conduct a systematic analysis.

In the present study, using 5′-leader nucleotide sequences and amino acid sequences translated in the three reading frames as queries to search public databases, we document the presence of intragenomic rearrangements involving segments of the 5′-leader sequence in geographically and temporally diverse isolates of SARS-CoV-2. The intragenomic rearrangements could modify the carboxyl-termini of the ORF8 (also in *Rhinolophus* bat *Sarbecovirus* β-CoVs) and ORF7b proteins; the serine-arginine-rich region of the nucleocapsid protein, generating the well characterized R203K/G204R paired mutation; and two sites of the NiRAN domain of the RdRp (nsp12).

Beyond SARS-CoV-2, we found similar rearrangements of 5′-UTR leader sequence segments including the TRS-L in all subgenera of β-CoVs except for *Hibecovirus* (possibly secondary to the availability of only 3 sequences in GenBank). These rearrangements are in the intergenic region between ORFs 3 and 4a, and at the distal end of ORF4b of the *Merbecovirus* MERS-CoV; intergenic regions in the *Embecoviruses* hCoV-OC43 (between S and Ns5) and hCoV-HKU-1 (between S and NS4); and in the distal end that encodes the Y1 cytoplasmic tail domain of nsp3 of *Nobecoviruses* of African *Rousettus* and *Eidolon* bats. We also found intragenomic rearrangements in α-CoVs in nsp2 (*Luchacovirus* subgenus), nucleocapsid (*Nyctacovirus* subgenus), and ORF5b or ORF4b (*Decacovirus* subgenus). No rearrangements involving 5′-UTR sequences were detected for the β-CoV SARS-CoV-1; the other 12 subgenera of α-CoVs including hCoV-229E and hCoV-NL63 infecting humans; or δ (*Andecovirus*, *Buldecovirus*, and *Herdecovirus* subgenera) and γ CoVs (*Brangacovirus*, *Cegacovirus*, and *Igacovirus* subgenera) for which wild birds are the main reservoir [12, 40].

The present study highlights an intragenomic source of variation involving duplication, inversion (in two α-CoVs subgenera) and translocation of 5′-UTR sequences to the body of the genome with potential implications on gene expression and immune escape of α- and β-CoVs in humans and bats causing mild-to moderate or severe disease in endemic, epidemic, and pandemic settings. Genome-wide annotations had revealed 1516 nucleotide-level variations at various positions throughout the entire SARS-CoV-2 genome [41] and a recent study documented outspread variations of each of the six accessory proteins across six continents of all complete SARS-CoV-2 proteomes which was suggested to reflect effects on SARS-CoV-2 pathogenicity [42]. However, the function and even expression of some of these accessory proteins remains a matter of debate due to inconsistencies derived from the use of bioinformatics predictions, and studies in different cell types and not in in vivo infection settings. The intragenomic rearrangements involving 5′-UTR sequences described here, which in several cases affect highly conserved genes with a low propensity for recombination, may underlie the generation of variants homotypic with those of concern or interest and with potentially differing pathogenic profiles.

## Methods

### Detection of 5′-UTR sequences in SARS-CoV-2 and SARS-CoV-related viruses in GenBank

To assess the presence of 5′-UTR sequence insertions in the body of the genome, we used 5- to 10-amino acid stretches from the three reading frames of the translated 5′-UTR nucleotide sequence of SARS-CoV-2 (Wuhan reference, NC_045512) as query sequences to search the GenBank^®^ database using the Basic Alignment Search Tool (BLAST)P^®^ (Protein BLAST: search protein databases using a protein query (nih.gov); [43]) for SARS-CoV-2 and SARS-CoV-related viral proteins encoding similar stretches. All nonredundant translated CDS + PDB + SwissProt + PRF excluding environmental samples from WGS projects were searched specifying severe acute respiratory syndrome coronavirus 2 as organism.

Using the accession number listed in PubMed (SARS-CoV-2 Resources—NCBI (nih.gov)) for the viral protein sequence, we obtained the respective nucleotide sequence and translated it using the insilico (DNA to protein translation (ehu.es) [44] and Expasy (ExPASy—Translate tool [45]) tools to determine by manual inspection and the BLASTN program [46] if the nucleotide sequences encoding said stretches were identical to those in the 5′-UTR nucleotide sequence of SARS-CoV-2 or SARS-CoV-related viruses.

Using nucleotide sequences instead of translated amino acid sequences from the 5′-UTR in the three reading frames as query sequences was unproductive to detect insertions in SARS-CoV-2 because of the large number of SARS-CoV-2 sequences in the GenBank database and the limit of 5000 results in the BLAST algorithm settings which yielded solely 5′-UTR sequences.

### Detection and validation of 5′-UTR sequences in regions other than the 5′-UTR of SARS-CoV-2 and SARS-CoV-related viruses in other databases

To detect isolates with similar insertions whose sequences had not been included in GenBank, we then searched the Global Initiative on Sharing All Influenza Data (GISAID) EpiFlu™ database of SARS-CoV-2 sequences (GISAID—Initiative; [47–49]) using as queries the nucleotide sequences of the insertions plus adjoining 20 nucleotides on either side from the viral genomes. This approach is limited by the fact that maximum number of search results in GISAID is 30. Information on location and timing of isolate collection was obtained from the GenBank and GISAID databases.

### Detection of 5′-UTR sequences in regions other than the 5′-UTR in coronaviruses other than SARS-CoV-2 and SARS-CoV-related viruses

We used the Rfam database (http://rfam.xfam.org/covid-19) with the curated Stockholm files containing UTR sequences, alignments and consensus RNA secondary structures of major genera of *Coronoviridae*; the representative RefSeq sequences for each genus obtained from the International Committee on Taxonomy of Viruses (ICTV) taxonomy Coronaviridae Study Group [2]); the reference sequences in the GenBank database; and listings in publications involving phylogenetic analyses of alpha-, delta-, and gamma-coronaviruses from NCBI Taxonomy [34, 50] to derive the 5′-UTRs of various CoVs.

We then utilized the 5′-UTR segments as query sequences to search for insertions in their respective genomes (nucleotide collection [nr/nt]; expect threshold: 0.05; mismatch scores: 2, − 3; gap costs: linear). The GSAID database does not include sequences of CoVs other than SARS-CoV-2 and therefore could not be used for this analysis.

If the intragenomic rearrangement detected using the 5′-UTR sequences involved a coding region, we translated the 5′-UTR insertion and adjacent segments using the insilico (DNA to protein translation (ehu.es) [44] and Expasy tools [45].

### Localization and sorting of intragenomic rearrangements

In terms of the locations of the insertions in the body of the genomes, the boundaries of nonstructural, structural, and accessory open reading frames were determined based on GenBank annotation and from manual inspection of multiple alignments and sequence similarities.

### Sorting and collection of further information on viral isolates with intragenomic rearrangements

In the results presented, we excluded matches to entries corresponding to the 5′-leader sequences in mRNAs from full viruses or defective interfering RNA particles, as well as protein sequences with > 80% unknown amino acids (represented by the letter X) in GenBank. The Supplementary section includes the accession numbers and collection site and date, and in some cases the SARS-CoV-2 lineages, for the isolates with intragenomic rearrangements involving 5′-UTR sequences.

### Detection of possible intragenomic rearrangements involving 3′-UTR sequences

We also searched for intragenomic rearrangements involving 3′-UTR sequences using the same approaches and datasets described for the 5′-UTR ones.

### Visualization of RNA secondary structures in segments with intragenomic rearrangements

RNA secondary structures of the 5′-UTR sequence insertion and adjacent sequences of the intragenomic rearrangement were visualized using forna, a force directed graph layout (ViennaRNA Web services; [51]), and the optimal secondary structures and their minimal free energies were determined using the RNAfold webserver [52, 53].

## Results

Using the approaches described in the Methods section, we conducted a systematic analysis of SARS-CoV-2 and other coronaviruses and detected insertions involving 5′-UTR sequences at various locations in β- and α-CoVs, as described below by subgenus.

### Intragenomic rearrangements at the distal end of ORF8 and ORF7b (Sarbecoviruses)

**Fig. 1:**
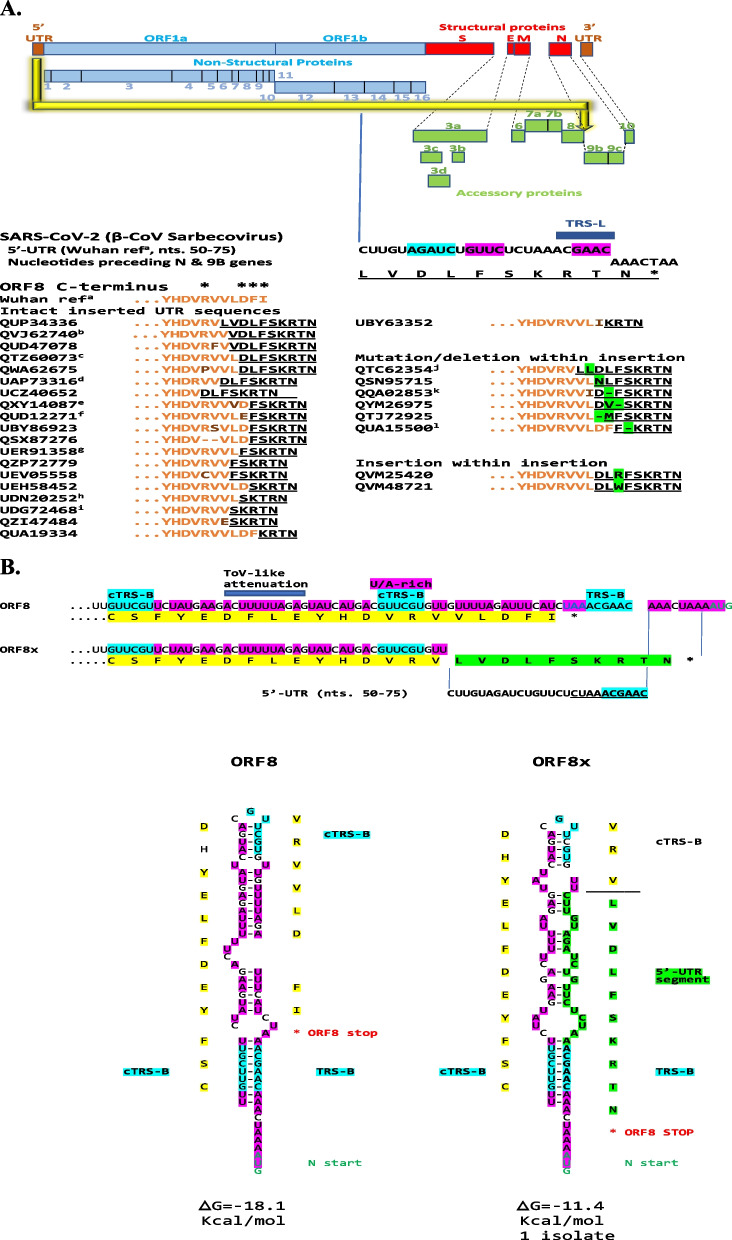
Modified carboxyl-termini of ORF8 protein predicted to be encoded by 5′-UTR sequence insertions in SARS-CoV-2. **A**. The largest 5′-UTR segment that was duplicated and translocated as an insertion to the carboxyl terminus of ORF8 is shown at the nucleotide and amino acid levels (latter underlined). All translocated 5′-UTR nucleotide sequence segments include TRS-L (dark blue box) with variable extents of SL3 (blue) and SL2 (red). Examples are shown, and corresponding similar sequences in GenBank as of February 20, 2022, are listed in the Additional file 1: legend to Fig. S1. The C-terminus of ORF8 in the Wuhan reference strain is depicted using orange letters with mutations in ochre; the asterisks over the C-terminus sequence designate residues contributing to the covalent dimer interface (Arg115, Asp119, Phe120, Iso121; [80]). The 5′-UTR insertions are shown as underlined letters in black with mutations, deletions, and insertions within them highlighted in green. **B**. Secondary structures of ORF8 RNA in reference strain and in that with longest 5′-UTR sequence intragenomic rearrangement. Nucleotide and amino acid sequences of the carboxyl termini of ORF8 from Wuhan reference (NC_0445512) and from isolate QUP34336 (USA/Minnesota, 2021-04-05) with the longest 5′-UTR sequence. ORF8 protein from the latter has modified carboxyl terminus and is therefore designated ORF8x. Amino acid sequence from the reference strain is highlighted in yellow while that encoded by the duplicated and translocated 5′-UTR segment is highlighted in green. Stop codon of ORF8 protein is depicted with a red asterisk, and initiation codon of N is in green letters. TRS-B core sequence and complementary TRS-Bs in *ORF8* and in *ORF8x* are highlighted in blue; and the uridine/adenosine tracks, including the torovirus-like attenuation sequence [56] are highlighted in fuchsia. 5′-UTR nucleotide and predicted translated amino acid sequences in ORF8X are in bold letters and highlighted in green

**Fig. 2:**
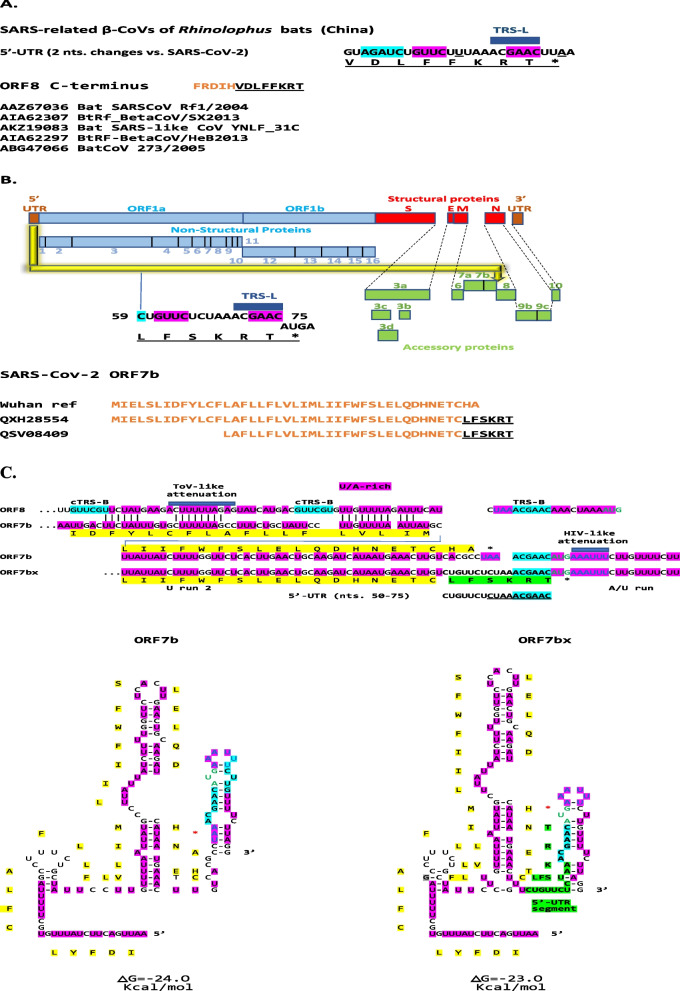
**A**. Modified carboxyl-termini of ORF8 predicted to be encoded by an insertion of a 5′-UTR segment in SARS-related β-coronaviruses of *Rhinolophus* bats from China. For SARS-related bat β-CoVs (BatSARSCoV Rf1/2004 and Bat CoV 273/2005 are subgroup 2b; [7]), all inserted terminal sequences were the same. The nucleotide sequence of the inserted 5′-UTR segment differed from that of SARS-CoV-2 by two nucleotides: a C to U change (underlined) which translates into an amino acid change (serine [S] to phenylalanine [F]), and a U to A (underlined) which introduces a stop codon. **B**. Modified carboxyl termini of ORF7b protein predicted to be encoded by an insertion of a 5′-UTR segment in SARS-CoV-2. The two isolates with predicted modified ORF-7 proteins are QXH28554 (USA/Alabama, 2021/04/14), and QSV08409 (USA/California; 2021/02/26); the latter has a truncated *ORF7b* and the former a truncated *ORF8*. Color codes and abbreviations are as in Fig. 1. **C**. Secondary structures of *ORF7b* and *ORF7bx* RNAs. Color scheme is as in Fig. 1B. An HIV-like attenuation sequence [57] is also highlighted

We found a U.S. isolate of SARS-CoV-2 in which a segment encompassing nucleotides 50–75 of the 5′-UTR was duplicated and translocated to the end of the accessory *ORF8* gene giving rise to a predicted ORF8 protein with modified carboxyl-terminus encoded by the translocated 5′-UTR sequences. Figure 1 summarizes the results of our systematic search which revealed 240 similar insertions of various lengths of the same 5′-UTR sequence at various points in a stretch of 7 amino acids (_115_RVVLDFI_121_) of the carboxyl-terminal sequence of the predicted ORF8 protein. As depicted in Additional file 1: legend to Fig. S1, these internal rearrangements were detected in temporally and geographically diverse isolates, collected from March 2020 to December 2021 in 38 USA states, Bahrain, China, Kenya, and Pakistan, which is not exhaustive of what exists. All translocated 5′-UTR nucleotide sequence segments include TRS-L with variable extents of SL3 and SL2, that could affect expression of the nucleocapsid gene located immediately after the *ORF8* gene [19], and all insertions alter the carboxyl-termini of predicted ORF8 proteins. The analysis also revealed that the insertions in some isolates had further changes involving point mutations, deletions, and insertions. Moreover, as shown in Fig. 2A, a similar 5′-UTR sequence insertion at the distal end of *ORF8* is seen in five *Sarbecovirus* β-CoVs from what is considered the animal reservoir for SARS-CoV-2, the *Rhinolophus* (horseshoe) bats residing in Indochina and Southwest China [54] all the way to England [55].

Figure 1C depicts the predicted secondary structure of ORF8 RNA without and with (ORF8x) the 5′-UTR sequence insertion. Both structures have similar predicted minimum free energy. The insertion involves the TRS-B sequence located in the intergenic region between *ORF8* and *N* genes and is preceded by a uridine-adenosine (U/A)-rich region including a sequence similar to the torovirus attenuation sequence [56], which like TRS-B, might cause the RNA-dependent RNA polymerase to pause, thereby facilitating the intragenomic rearrangement as it is theorized to do during subgenomic RNA synthesis. Additional file 1: Fig. S2A shows the predicted RNA structures of the most common ORF8x variants with similar predicted minimum free energy, while Additional file 1: Fig. S2B shows an alternative RNA structure involving the interaction between the TRS-B in the intergenic region with a second and closer complementary TRS-B, yielding a similar predicted minimum free energy.

A shorter segment of the SARS-CoV-2 5′-UTR leader sequence (nts. 57–95, including TRS-L and SL3) than that described for *ORF8* insertions was also duplicated and translocated to the end of *ORF7*b in two SARS-CoV-2 isolates (Fig. 2B), one with a truncated ORF7b and the other with a truncated *ORF8*, which may have favored the internal rearrangements. Figure 2C shows the predicted secondary structures of the region with the intragenomic rearrangement, and as that of ORF8, involves the intergenic TRS-B sequence which is preceded and followed by a U/A-rich region, in this case also incorporating an HIV-like attenuation sequence (AAAUUU; [57]. Figure 2C also shows a region of similarity between *ORF8* and *ORF7b* which precedes the intragenomic rearrangement.

### Intragenomic rearrangements at the end of the segment encoding the serine-arginine-rich region of the N protein (SARS-CoV-2)

**Fig. 3:**
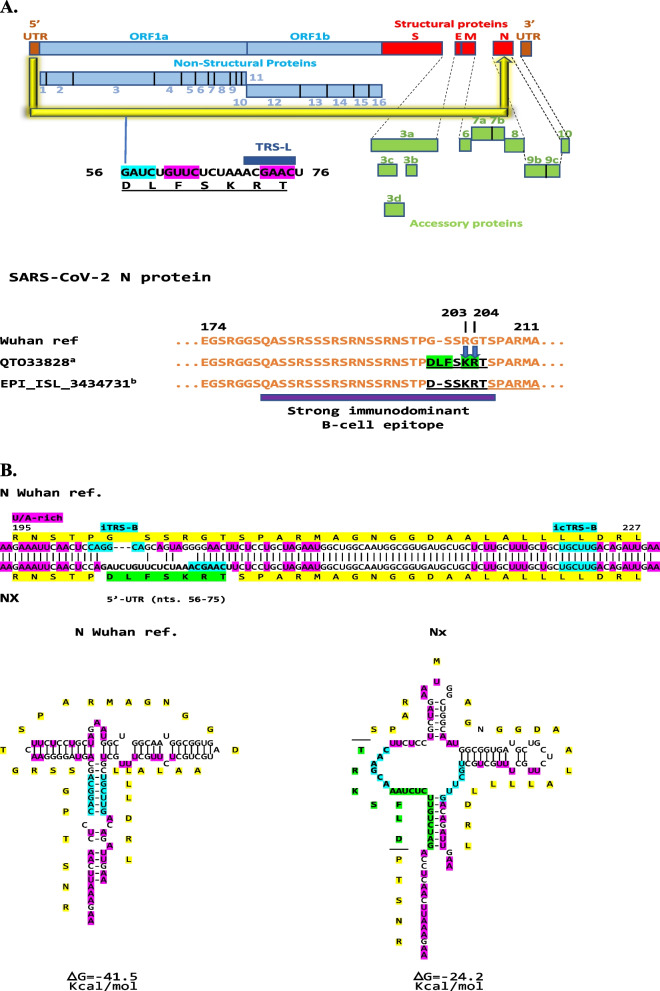
**A**. Insertion of 5′-UTR segment into the nucleotide segment encoding the serine-arginine-rich region of the nucleocapsid (N) in SARS-CoV-2. The R203K and G204R amino acid substitutions (blue arrows) which are commonly present concomitantly are encoded in this case by the insertion of a 5′-TR segment into the serine-arginine (SR)-rich region of the N protein at the end of a strong immunodominant B-cell epitope (purple box; [105]). Examples of isolates (a. and b.) with 5′-UTR sequences are provided in the figure and a full listing is provided in the Additional file 1: legend to Fig. S3. **B**. Secondary structures of *N* and *Nx* RNAs. Color scheme is as in preceding figures

In terms of structural proteins of SARS-CoV-2, we found a similar segment of the 5′-UTR corresponding to the leader sequence (nucleotides 56–76 of the Wuhan reference strain [NC_045512], including TRS-L, SL3 and part of SL2, and encoding the 7-amino acid sequence DLFSKRT) within the *N* gene at the end of the nucleotide segment encoding the serine-arginine region, as exemplified by isolate QTO33828 (USA/Texas, Fig. 3A). The 5′-UTR segment changes 5 of 7 positions, including R203K/G204R, which are known to be frequent co-occurring mutations in the N protein; however, the rest of the N protein sequences are well conserved with only 1 or 2 amino acid differences in the isolates identified. In another set of SARS-CoV-2 isolates, as exemplified by isolate EPI-ISL_3434731 (Brazil/Espirito Santo) in Fig. 3A, the same 5′-UTR sequence is present in *N* but without the predicted translated leucine (L) residue and the phenylalanine (F) changed to serine (S), more closely approaching the Wuhan reference strain sequence.

The predicted RNA structures of *N* without and with (*Nx*) the 5′-UTR sequence insertions are shown in Fig. 3B, with that for Nx being less stable with almost half the minimum free energy. Although there is no TRS-B in the N region where the intragenomic rearrangement was found, there is an inverted TRS-B that can pair with a complementary inverted TRS-B, both surrounded by U/A-rich regions which could facilitate the intragenomic rearrangement.

In total, 37 SARS-CoV-2 isolates had 5′-UTR sequences in their *N* gene, in contrast to ~ 336,000 isolates with either R203K or G204K as per NCBI Virus (mutations in SARS-CoV-2 SRA data); most were isolates of the variant of concern gamma GR/501Yv3 (P1) lineage (first detected in Brazil and Japan) from Brazil, Chile, and Peru, but also alpha (B.1.1.7; first detected in Great Britain) from USA and Canada (Additional file 1: legend to Fig. S3). The R203K/G204R co-mutation has been associated with B.1.1.7 (alpha) lineage emergence, which along with variants with the co-mutation including the P1 (gamma) lineage [58], possess a replication advantage over the preceding lineages and show increased nucleocapsid phosphorylation, infectivity, replication, virulence, fitness, and pathogenesis as documented in a hamster model, human cells, and COVID-19 patients including an analysis of association between COVID-19 severity and sample frequency of R203K/G204R co-mutations [59–61]. The intragenomic rearrangement in N might be one rare way for SARS-CoV-2 to acquire the R203/G204K co-mutation.

### Intragenomic rearrangements in the region encoding the Nidovirus RNA-dependent RNA polymerase associated nucleotidyl transferase (NiRAN) domain (SARS-CoV-2)

**Fig. 4:**
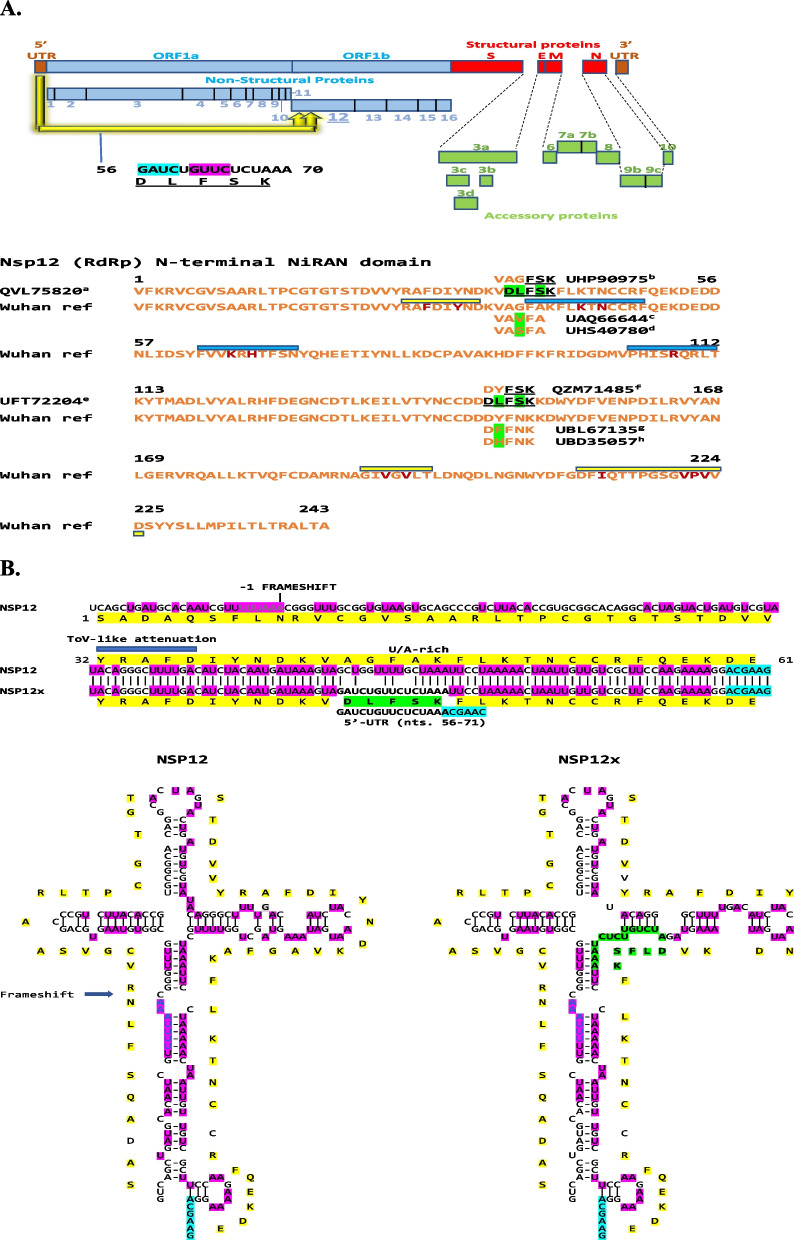
**A**. Insertions of a 5′-UTR sequence into two sites within the nucleotide segment encoding the *Nidovirus* RdRp associated nucleotidyl transferase (NiRAN) domain of the RNA-dependent RNA polymerase (nsp12) of SARS-CoV-2. Examples of isolates with 5′-UTR sequences at the proximal and distal sites are provided in the figure and a full listing is provided in the Additional file 1: legend to Fig. S4, as is a listing of variants with single amino acid changes relative to the Wuhan reference strain in the segment corresponding to the insertion. The Wuhan reference strain sequence corresponding to the insertion areas is the most abundant among SARS-CoV-2 isolates. The nsp12-nsp9 interface regions are shown with yellow bars and key residues therein with ochre letters, while the contact regions with GDP are indicated with blue boxes and key residues therein in ochre. **B**. Secondary structures for RNAs in the proximal sites in *nsp12* and *nsp12x*. Color scheme is as in previous figures. The site for -1 frameshifting is also highlighted

Another example of intragenomic rearrangement is the presence of the translated sequence (DLFSK) of a shorter segment of 5′-UTR sequence (nts. 56–70 in Wuhan reference strain, including parts of SL2 and SL3 but not TRS-L) at amino acids 36–40 of the NiRAN domain of the viral RdRp (nsp12) in isolates QVL75820 (EPI_ISL_1209225, USA/Seattle, 2021-03-28; lineage: B.1.2 [Pango v.3.1.20 2022-02-02]) and EPI_ISL_1524008 (USA/Washington, 2021-03-28; VOC Alpha GRY (B.1.1.7 + Q.*) first detected in the UK) and at amino acids 146–150 in isolates UFT72204 (EPI_ISL_6912949, USA/Colorado, 2021-10-27; VOC Delta GK [B.1.617.2 + AY.*] first detected in India), EPI_ISL_1384819 (India/Maharashtra, 2021-02-12; lineage: B.1.540 [Pango v.3.1.20 2022-02-02]) and EPI_ISL_1703925 (India/Maharashtra, 2021-02-07; B.1.540 lineage), respectively (Fig. 4A). The latter strains have only one amino acid change outside of the insertions relative to the Wuhan reference strain. A subsegment of 5′-UTR (nts. 62–70) translated as FSK is present at the more proximal site (amino acids 38–40) in 230 isolates isolated from diverse populations at various times (listed in Additional file 1: legend to Fig. S4) and exemplified by isolate UHP90975 [USA/Wisconsin, 2021-12-13] in Fig. 4A. Isolate QZM71485 (USA/New York, 2021-08-05) exemplifies isolates with the FSK sequence at the more distal site (amino acids 148–150). Examples of the most common single amino acid changes in overlapping segments of other isolates are listed as comparators, and they have similar or lower frequency than those of the 5′-UTR segments (summarized in Additional file 1: Table S1). However, the Wuhan reference strain sequence corresponding to the areas with 5′-UTR sequences is the most abundant among SARS-CoV-2 isolates.

Genes encoding components of the replication-transcription complex, such as the RdRp (nsp12) [62, 63], are highly conserved and have a low propensity for recombination among CoVs [34]. The nsp12 NiRAN domain is one of the five replicative peptides that are common to all Nidovirales and used for species demarcation because it is not involved in cross-species homologous recombination [64]. However, as in other examples here of conserved genes, it is involved in intragenomic rearrangements of 5′-UTR sequences. Figure 4B shows the predicted structure RNA structures for the proximal site of intragenomic rearrangement in nsp12 and nsp12x (with 5′-UTR sequence). As in the case of the example in the intragenomic rearrangement in N there is no TRS-B at the site of intragenomic rearrangement, which is however preceded by a sequence similar to the torovirus-like attenuation sequence within a U/A-rich region which may facilitate pausing of the RdRp.

The NiRAN domain of nsp12 is involved in the NMPylation of nsp9 [65] during the formation of the replication-transcription complex (interface regions [66] are shown with yellow bars and key residues therein with ochre letters in Fig. 4). The 5′-UTR sequence at the proximal site in the nsp12 NiRAN domain overlaps with one of the interface regions with nsp9 but does not affect key interface residues or alter the charge distribution of amino acid side chains in the overlap region. The nsp12 NiRAN domain also exhibits a kinase/phosphotransferase like activity [67], is involved in protein-primed initiation of RNA synthesis [68] and catalyzes the formation of the cap core structure (GpppA; contact regions with GDP [66] indicated with blue boxes and key residues therein in ochre in Fig. 4A) [69]. The 5′-UTR sequence at the proximal site in nsp12 NiRAn domain is close to the first contact region with GDP.

### Intragenomic rearrangements in β-CoVs of Merbecovirus, Embecovirus, and nob$ecovirus subgenera

#### Merbecovirus

**Fig. 5:**
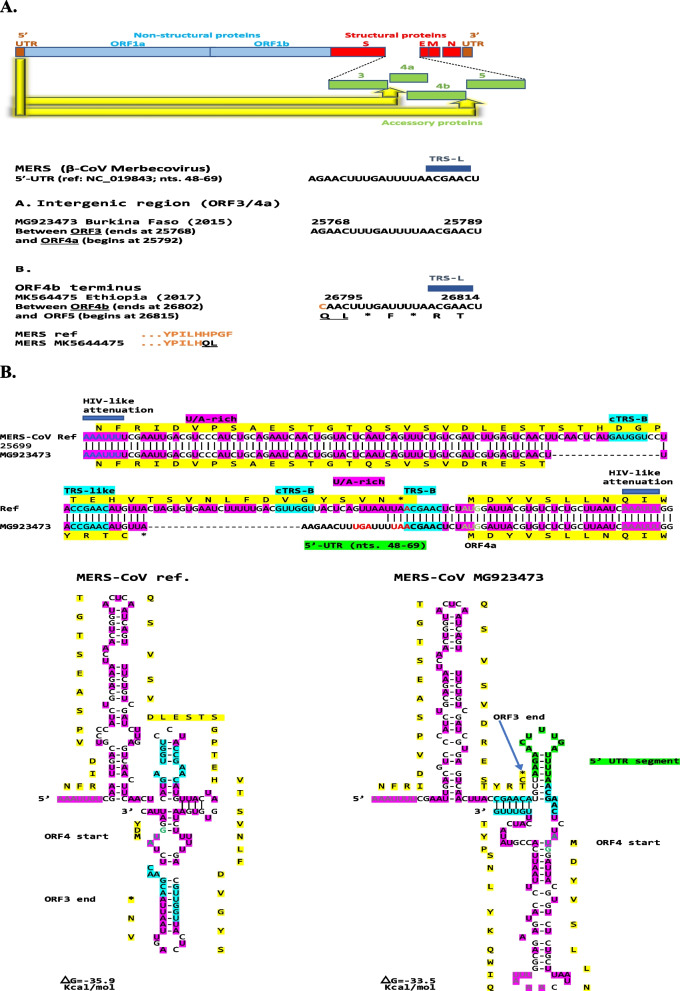
**A**. Intragenomic rearrangement with 5′-UTR sequences present in the intergenic regions between genes encoding p3 and 4a as well as between those encoding p4b and p5 of the *Merbecovirus* Middle East respiratory syndrome (MERS)-CoV. A segment of the 5′-UTR of the MERS-CoV including TRS-L and part of the second of the two stem-loops is present in the intergenic region between genes encoding p3 and p4a in isolate MG923473 (Burkina Faso, 2015) and between those encoding p4b and p5 affecting the predicted carboxyl-terminal end of ORF4b in isolate MK564475 (Ethiopia, 2017). In the latter case, the last 4 amino acids (HPGF) of ORF4b in the reference MERS-CoV sequence (NC_019843) are replaced by two amino acids (QL). The Q residue is encoded by a cytosine present in the reference sequence (indicated in orange color) and two adenosines incorporated by the 5′-UTR sequence. **B**. RNA secondary structures of the intergenic region between genes encoding p3 and p4a in MERS-CoV without and with intragenomic rearrangement. Color scheme is as in previous figures

**Fig. 6:**
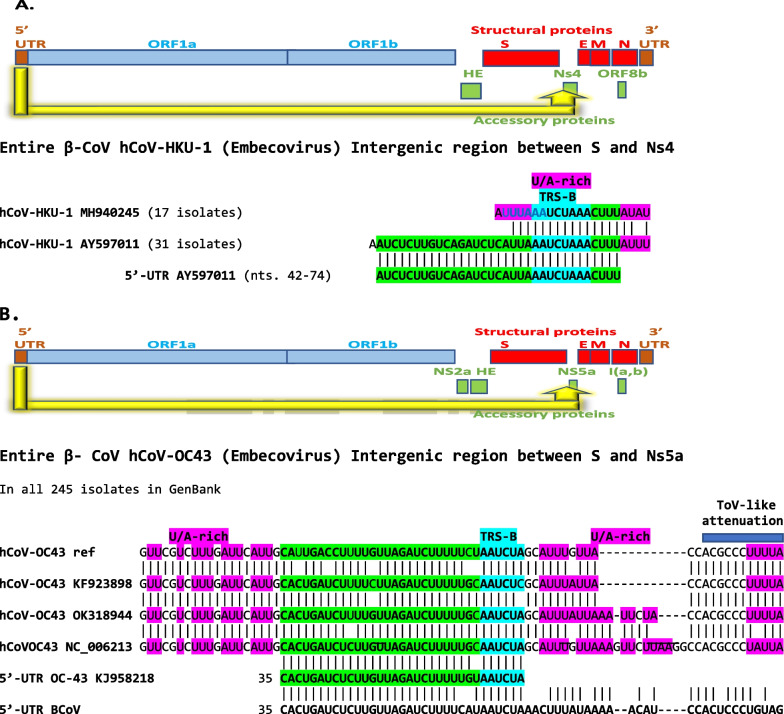
**A**. Presence of hCoV-HKU-1 (β-CoV *Embecovirus*) of 5′-UTR sequence in the intergenic region between the spike (*S*) and the *Ns4* genes. The hCoV-OC43 5′-UTR sequence inserted is identical to that of bovine coronavirus (BCoV) 5′-UTR (shown at the bottom of the figure) except for one nucleotide (an adenosine [A] instead of a guanosine [G] in BCoV). 31 out of 48 variants (65%) in GenBank have this intragenomic rearrangement. **B**. Presence in in the intergenic region between the *S* and *Ns5a* genes hCoV-OC43 (β-CoV *Embecovirus*) of sequences of various lengths of the same 5′-UTR region. As in the case of hCoV-HKU1, the 5′-UTR segment translocated to the intergenic region between *S* and *Ns5a* of hCoV-OC43 variants is similar to that of BCoV. Differences among them (all 245 isolates in GenBank) are distally to the TRS-B and involve various extents of sequences similar to the 5′-UTR of BCoV

As shown in Fig. 5A, a segment of the 5′-UTR of the β-CoV *Merbecovirus* MERS-CoV including TRS-L and part of the second of the two stem-loops is present in the intergenic region between the genes encoding p3 and p4a in isolate MG923473 (Burkina Faso, 2015) and at the distal end of the gene encoding p4b in isolate MK564475 (Ethiopia, 2017). In the latter case, the last 4 amino acids (HPGF) of p4b in the reference MERS-CoV sequence (NC_019843) are predicted to be replaced by two amino acids (QL). The Q residue is encoded by a cytosine present in the reference sequence (indicated in orange in Fig. 6A) and two adenosines incorporated by the 5′-UTR sequence. Figure 5B depicts the predicted RNA secondary structures without and with the insertion corresponding to the intragenomic rearrangement between the genes encoding p3 and p4b. The structures have similar predicted minimum free energy, and the rearrangement involves the intergenic TRS-B sequence which is preceded and succeeded by torovirus-like attenuation sequences. It is unknown whether these sequences, which function as attenuation sequences in other viruses, are functional or simply secondary to the fact that AU-rich sequences are frequent in coronavirus genomes.

#### Embecoviruses

The β-CoV *Embecovirus* hCoV-HKU1 is a sister taxon to murine hepatitis virus and rat sialodacyoadenitis virus [70]. Out of 48 HKU-1 isolates in GenBank, a 5′-UTR sequence including TRS-L, SL3 and most of SL2 (nucleotides 42–74 in hCoV-HKU-1 references NC_006577 and AY597011) is present in 31 isolates (65%) between the *S* and *Ns4* genes (Fig. 6A). The hCoV-HKU-1 NS4 protein is structurally similar to the hCoV-OC43 ns5a protein whose function is detailed in the Discussion section.

All 245 full genome isolates of the β-CoV *Embevovirus* hCoV-OC43 in GenBank had 5′-UTR-leader sequences (largest spanning nucleotides 35–67 of the hCoV-OC43 reference strain KJ958218) between the spike (*S*) and *Nsp5a* genes (Fig. 6B). The insertions did not affect the protein sequences of either S or Nsp5a. The hCoV-OC43 5′-UTR sequence inserted is identical to that of bovine coronavirus (BCoV) 5′-UTR except for one nucleotide (an adenosine substituted by a guanosine in BCoV) up to the TRS-B, and sequences of varying length after the TRS-B show similarities to BCoV 5′-UTR, which is consistent with a most probable bovine or swine coronavirus origin for hCoV-OC43 [71]. The 5′-UTR sequence insertion sequence is also present in a molecularly characterized cloned hCoV-OC43 *S* gene [72].

#### Nobecoviruses

**Fig. 7:**
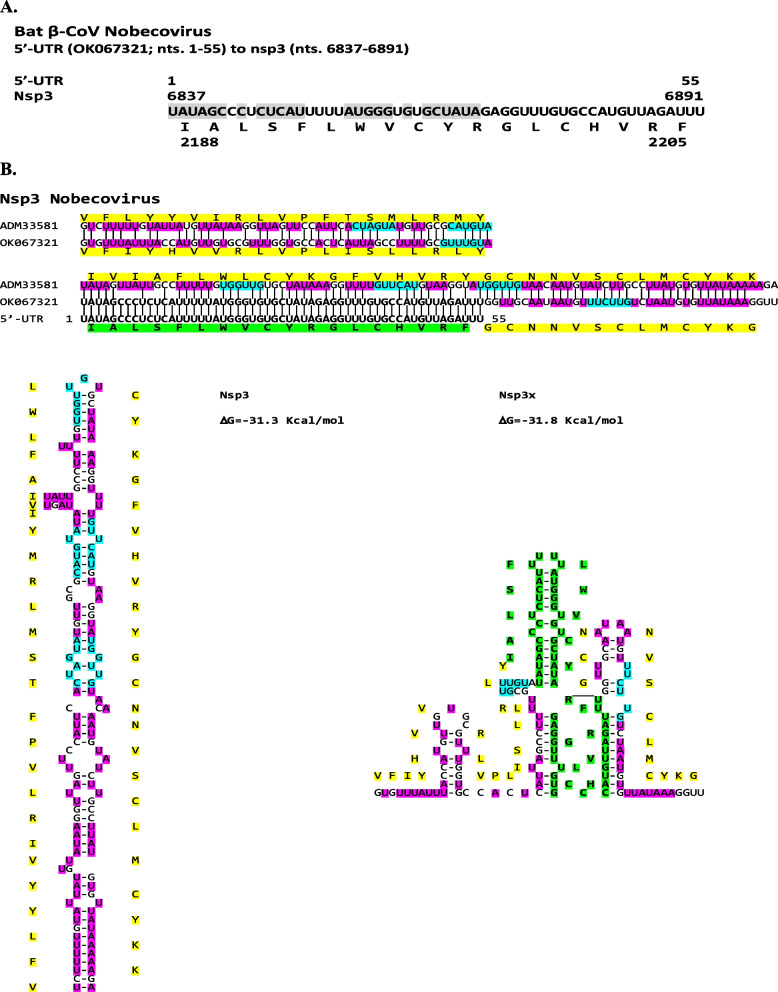
**A**. Presence of 5′-UTR sequence in the Bat β-CoV *Nobecovirus nsp3* gene. **B**. Secondary structures of *nsp3* and *nsp3x* RNAs. Color scheme is as in previous figures

An intragenomic rearrangement involving a 5′-UTR sequence (nucleotides 1–55) to distal end encoding the C-terminal cytoplasmic Y1 domain of nsp3 (nucleotides 6837–6891; amino acids 2188–2205), is seen in the β-CoV subgenus *Nobecovirus* of African bats, namely isolates MIZ240 (OK067321) and MIZ178 (OK067320) from *Rousettus madagascariensis* bats and isolates CMR900 (MG693169; protein: AWV67046), CMR705-P13 (MG693172, protein: AWV67070), and unclassified (NC_048212) from *Eidolon helvum* bats (Cameroon). Using the translated nucleotide sequence as query, the following additional isolates were detected: *Eidolon helvum* (Cameroon) isolates CMR704-P12 (YP_009824989 and YP_009824988), and CMR891-892 (AWV67062). The 5′-UTR sequence involved in this intragenomic rearrangement does not include the TRS-L and includes a stem-loop structure highlighted in grey in Fig. 7A. The position of the translated sequence of the 5′-UTR identical sequence is amino acids 2188–2205, which corresponds to amino acids 1567–1584 in SARS-CoV-2 nsp3. Figure 7B depicts the predicted secondary structures of *nsp3* and *nsp3x* RNAs with the intragenomic rearrangement. Both structures have similar predicted minimum free energy. Although there are no TRS-B sequences present in this region the rearrangement takes place adjacent to an inverted complementary TRS-B within a U/A-rich region.

### Intragenomic rearrangement in nsp2 of rodent α-CoVs subgenus Luchacovirus

**Fig. 8:**
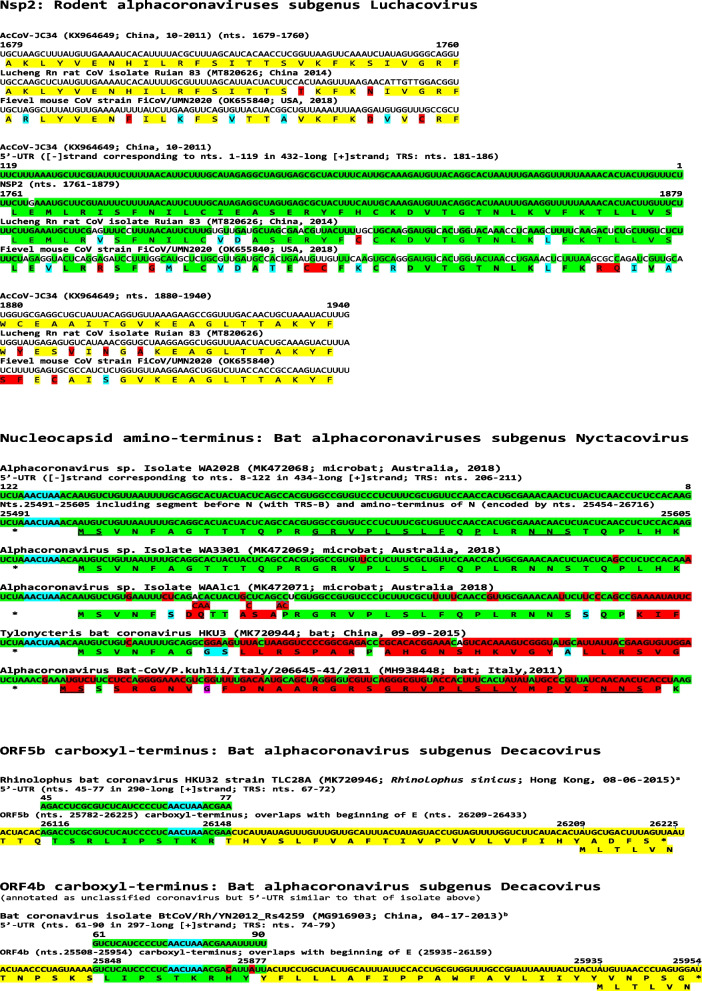
Intragenomic rearrangement in *nsp2* of rodent alphacoronaviruses subgenus *Luchacovirus*, *N* of bat alphacoronaviruses subgenus *Nyctacovirus* (A) and *ORF5b* or *ORF4b* of bat alphacoronaviruses subgenus *Decacovirus*. Nucleotide and amino acid sequences of intragenomic rearrangements are shown. 5′-UTR sequence (negative strand) is highlighted in green. Conservative amino acid substitutions are highlighted in blue while non-conservative ones are highlighted in red. For the intragenomic rearrangement in *nsp2* of rodent alphacoronaviruses subgenus *Luchacovirus*, two examples of isolates (listed by rodent of origin) with *nsp2* nucleotide sequences up to 75% similar to the 5′-UTR sequences are shown. There appears to be a temporal gradient with the most similar sequence (99%) in isolate KX964649 (China, 2011-10) to the least similar (59%) in isolate OK655840 (USA, 2018). The temporal gradient holds within animals from the same genus, which would suggest that the translocated sequence is the oldest and the rest reflect more recent mutations. For the predicted secondary structures of the RNAs corresponding to the intragenomic rearrangement and adjacent sequences, the minimum free energy increases among variants from those with the most to those with the least similar sequence to the 5′-UTR insertion (Additional file 1: Fig. S3). Color scheme is as in previous figures

As shown in Fig. 8A, a segment of the 5′-UTR (nts. 1–119) of the *Luchacovirus* AcCoV-JC34 (KX964649; isolated in China, 2011-10 from the rodent *Apodemus chevieri*) was duplicated, inverted, and translocated to the genomic region encoding the nonstructural protein nsp2 (nts. 1679-1760). The latter sequence in *nsp2* differs by only one nucleotide from that in the 5′-UTR (99% similarity), and it is also present with varying degrees of similarity in other rodents. Two examples are shown in Fig. 8A for isolates from rat (Lucheng Rn rat CoV isolate Ruian 83; MT820626; isolated from *Rattus norvegicus* in China, 2014, 76% similar), and mouse (Fievel mouse CoV strain FiCoV/UMN2020 (OK655840; isolated from *Mus musculus* in USA, 2018, 59% similar). Other isolates (listed by rodent of origin) with intragenomic rearrangements in *nsp2* with nucleotide sequences up to 75% similar to the 5′-UTR sequences include: *Apodemus chevrieri* (MT820625, China, 2015, 93% similar); *Apodemus agrarium* (MZ328302, China, 2016, 93% similar); *Eothenomys miletus* (MT820627, China, 2014, 81% similar); *Eothynomys melanogaster* (KY370054, China, 2015-12, 79% similar); *Myodes rufocanus* (KY370045, China, 2014-08, 79% similar); *Rattus losea* (KY370050, China, 2015-05, 78% similar); *Rattus norvegicus* (MK163627, United Kingdom, 2014-06-23, 78% similar; NC_032730, China, 2013; MT549854, China, 2016-12, 76% similar; MW802582, China, 2017-03-07, 76% similar); and *Brylmys bowersi* (MZ328301, China, 2016, 77% similar).

There appears to be a temporal gradient with the most similar sequence (99%) in isolate KX964649 (China, 2011-10) to the least similar (59%) in isolate OK655840 (USA, 2018). The temporal gradient of decreasing similarity holds within rodents from the same genus, which would suggest that the translocated sequence is the oldest and the rest reflect more recent mutations. This is consistent with a possible common ancestor for all rodent α-CoVs sampled so far, with phylogenetic analyses suggesting relatively frequent host-jumping among the different rodent species [50]. The minimum free energy of the predicted RNA secondary structures of the intragenomic rearrangement and adjacent sequences increases from the most similar to the least similar to the 5′-UTR insertion (Additional file 1: Fig. S3). The function of the region of intragenomic rearrangement in nsp2 remains to be determined and it does not overlap with that contributing to inflammation via NF-κB activation in the α-CoV porcine transmissible gastroenteritis virus [73].

### Intragenomic rearrangements in N of bat α-CoVs subgenus Nyctacovirus

As shown in Fig. 8B, in this intragenomic rearrangement in bat α-CoVs subgenus *Nyctacovirus*, a 115-nucleotide-long segment of the 5′-UTR is duplicated, inverted (negative-sense strand) and translocated to the proximal end of the nucleocapsid gene thereby encoding the predicted first 38 amino acids of the amino-terminus of N. Other variants share the sequence with lesser similarity to the 5′-UTR sequence. There is a TRS-B sequence (AACUAA) at the beginning of the insertion, and the negative strand 5′-UTR sequence also has a AACUAA sequence, which may have mediated the intragenomic rearrangement.

### Intragenomic rearrangements in ORF5b/4b of bat α-CoVs subgenus Decacovirus

Orb5b and ORF4b proteins are 53% similar (including conservative substitutions) between bat alphacoronaviruses subgenus *Decacovirus* shown in Fig. 8C. In both sets of viruses, *ORF5b* or *ORF4b* overlap the beginning of the membrane (*M*) gene, i.e., there is no intergenic region between them and *M*. However, there is a TRS-B sequence (AACUAA) within the 3′ end of *ORF5b* and *ORF4b* where the intragenomic rearrangement occurs. Viruses with similar intragenomic rearrangements in *ORF5b* include: *Rhinolophus* bat coronavirus HKU32 strain TLC28A (MK720946), *Rhinolophus* bat coronavirus HKU32 strain TLC26A (MK720945; Hong Kong, 08-06-2005), Alphacoronavirus sp. strain bat/Yunnan/HcYN26/2020 (MZ081384; *Hipposideros cineraceus*; China, 07-29-2020), Alphacoronavirus sp. strain bat/Yunnan/RsYN12/2019 (MZ081386; *Rhinolophus sinicus*; China, 10-22-2019), Alphacoronavirus sp. strain bat/Yunnan/MmYN16/2020 (MZ081385; *Myotis muricola*; China, 04-18-2020), Alphacoronavirus sp. strain bat/Yunnan/RmYN21/2020 (MZ081387; *Rinolophus malayanus*; China, 06-03-2020). Viruses with similar intragenomic rearrangements in *ORF4b* include: Bat coronavirus isolate BtCoV/Rh/YN2012_Rs4259 (MG916903; China, 04-17-2013), Bat coronavirus isolate BtCoV/Rh/YN2012_Rs4125 (MG916902; China, 09-16-2012). The functional significance of this intragenomic rearrangement remains to be determined.

### Intragenomic rearrangements of 5′-UTR sequences were not detected in some β-or α-, or in any γ- and δ-CoVs, and no intragenomic rearrangements of 3′-UTR sequences were detected in any coronavirus

A listing of the other coronaviruses analyzed beyond the ones found to have intragenomic rearrangements is provided at the end of the Additional file 1. The directionality of potential translocation appears to be in the 5′–3′ direction as further underscored by the absence of 3′-UTR sequence insertions in any of the viruses analyzed.

## Discussion

We here describe intragenomic rearrangements involving 5′-UTR sequences and the coding section of the genome of beta- and alphacoronaviruses. Additional file 1: Fig. S4A summarizes the locations of insertions in accessory, structural, and nonstructural genes of SARS-CoV-2, which for at least the accessory and structural genes appear to involve and/or affect the template switching mechanism by creating new regions of homology for interaction with TRS-L. The presence of conserved complementary sequences (CCSs) in the 5′- and 3′-UTRs potentially involved in circularization of the genome during subgenomic RNA synthesis has been reported [74]. As shown in Additional file 1: Fig. S4B, the 5′-UTR sequences involved in intragenomic rearrangements in SARS-CoV-2 shown in the present work usually include the TRS-L and span approximately half of the 5′ CCS, thus potentially facilitating circularization of the genome from locations closer to the 3′-UTR. The 5′-UTR sequences involved in intragenomic rearrangements may also facilitate other long-distance RNA-RNA interactions contributing to the complex coronavirus transcription process [75].

Most of the 5′-UTR sequences duplicated and translocated include TRS-L. Extending the homology region of interaction between the TRS-L in the 5′-leader and the TRS-L introduced in a particular area of the body of the genome optimizes minimum free energy of the interaction. Such facilitation may favor expression of certain genes over that of others, thereby altering the hierarchy in gene expression. Because insertions are in various locations of viral genes, including some encoding nonstructural proteins, they may propitiate formation of new subgenomic RNAs thereby expanding the repertoire of proteins and even transforming noncanonical subgenomic messenger RNAs, i.e., not associated with TRS homology, to canonical ones. SARS-CoV-2 and other CoVs have been reported to generate noncanonical subgenomic RNAs in abundance, accounting for up to a third of subgenomic messenger RNAs in cell culture models of infection and increasing in proportion over time [76].

The structural genes control genome dissemination [63] while the accessory genes in the same region of the genome may be involved in adaptation to specific hosts, modulation of the interferon signaling pathways, the production of pro-inflammatory cytokines, or the induction of apoptosis [77], among other mechanisms underlying immune evasion and pathogenesis. Gaining insight into the effect of the amino acid changes introduced by the 5′-UTR sequences is likely to shed light into pathogenesis and immune evasion mechanisms. For instance, a few point mutations can have a profound effect as exemplified by the few mutations in the C-terminus of the spike protein that transform the feline CoV associated with mild disease to one, the feline infectious peritonitis virus, which is generally lethal [78].

*ORF8* had been postulated to originate from *ORF7a* by non-homologous recombination, and a predicted structure model of the ORF8 protein of SARS-CoV-2 revealed a ~ 60-residue core like that of SARS-CoV-2 ORF7a protein [79] with the addition of two dimerization interfaces, one covalent and the other noncovalent, unique to SARS-CoV-2 ORF8 [80]. In the C-terminus of ORF8 that would be predicted to be altered by 5′-UTR sequence insertions (i.e., _115_RVVLDFI_121_), R115, D119, F120, and I121 contribute to the covalent dimer interface (marked with asterisks in Fig. 1) with R115 and D119 forming salt bridges that flank a central hydrophobic core in which V117 interacts with its symmetry-related counterpart [80].

How the C-terminal insertions and changes therein affect the dimerization of ORF8 protein remains to be determined and described functions for ORF8 protein remain a matter of debate [81]. However, the predicted changes caused by insertions might contribute to immune evasion by SARS-CoV-2 by affecting the interactions of the ORF8 glycoprotein homodimer with intracellular transport signaling, leading to down-regulation of MHC-I by selective targeting for lysosomal degradation via autophagy [82], and/or extracellular signaling involving interferon-I signaling [83], mitogen-activated protein kinases growth pathways [84], the tumor growth factor-β1 signaling cascade [85] and interleukin-17 signaling promoting inflammation and contributing to the COVID-19-associated cytokine storm [86].

The carboxyl-terminal region of the ORF8 protein may include T- and/or B-cell epitopes that may be affected by the variations described. To this end, approximately 5% of CD4+ T cells in most COVID-19 cases are specific for ORF8 protein, and ORF8 protein accounts for 10% of CD8+ T cell reactivity in COVID-19 recovered subjects [87, 88]. Another possible effect of the insertions stems from the fact that anti-ORF8 protein antibodies are detected in both symptomatic and asymptomatic patients early during infection by SARS-CoV-2 [89] and diagnostic assays for SARS-CoV-2 infection that target only accessory genes or proteins such as ORF8 may be affected [39].

In terms of the potential consequences of intragenomic rearrangements involving *ORF7b* of SARS-CoV-2, the function of the SARS-CoV-2 ORF7b protein remains to be determined and has been suggested to mediate tumor necrosis factor-α-induced apoptosis based on cell culture data [90] and theoretically the dysfunction of olfactory receptors by triggering autoimmunity [91].

We also found intragenomic rearrangements in the nucleocapsid gene of SARS-CoV-2 and bat α-CoVs subgenus *Nyctacovirus*. The nucleocapsid is the most abundant protein in CoVs, interacts with membrane protein [92, 93], self-associates to provide for efficient viral assembly [94], binds viral RNA [95] and has been involved in circularization of the murine hepatitis virus genome via interaction with 3′- and 5′-UTR sequences which may facilitate template switching during subgenomic RNA synthesis [96]. Phosphorylation transforms N-viral RNA condensates into liquid-like droplets, which may provide a cytoplasmic-like compartment to support the protein’s function in viral genome replication [93, 97].

The phosphorylation-rich stretch encompassing amino acid residues 180–210 (SR region) encoded by the nucleotide segment where 5′-UTR sequences were detected in SARS-CoV-2, serves as a key regulatory hub in N protein function within a central disordered linker for dimerization and oligomerization of the N protein, which is phosphorylated early in infection at multiple sites by cytoplasmic kinases [97]. Serine 202 (numbering of reference Wuhan strain), which is phosphorylated by GSK-3, is conserved in the predicted translated 5′-UTR sequence next to the R203K/G204R co-mutation, as is threonine 205, which is phosphorylated by PKA [98, 99]. R203 and G204 mutations affect the phosphorylation of serines 202 and 206 in turn affecting binding to protein 14-3-3 and replication, transcription, and packaging of the SARS-CoV-2 genome [100–102].

The *N* gene displays rapid and high expression, high sequence conservation, and a low propensity for recombination [34, 103, 104]. However, it can show variation driven by internal rearrangement which does not affect the length of the protein. The N protein is highly immunogenic, and its amino acid sequence is largely conserved, with the serine-arginine (SR) region being a strong immunodominant B-cell epitope [105] as highlighted in Fig. 3A.

The functional significance of the intragenomic rearrangement in *N* of bat α-CoVs subgenus *Nyctacovirus* remains to be determined. Although in infectious bronchitis virus, the amino terminal domain of N protein has been shown to interact with nucleotide sequences in the 3′-UTR which is relevant to viral RNA packaging, the amino acids that are critical for such interaction are more distally located in the amino terminus (amino acids 76 or 94) [106, 107] than those encoded by the intragenomic rearrangement in this case.

The intragenomic rearrangements found in MERS-CoV may modulate immune evasion by bringing regulatory sequences to the intergenomic regions preceding the *4a* and *5* genes and modulating their expression. p4a, a double stranded RNA-binding protein, as well as p4b and p5 of MERS-CoV are type-I IFN antagonists [108–111]. p4a prevents dsRNA formed during viral replication from binding to the cellular dsRNA-binding protein PACT and activating the cellular dsRNA sensors RIG-I and MDA5 [110, 111]. p4a is the strongest in counteracting the antiviral effects of IFN via inhibition of both its production and Interferon-Stimulated Response Element (ISRE) promoter element signaling pathways [112]. The latter findings were obtained in cell cultures and studies in an in vivo infection are warranted. To this end, a more recent study associated p4b with inflammatory pathology and suppression of autophagy in murine lungs thereby highlighting the complex interplay of proteins during virus replication under in vivo physiological conditions [113].

Like SARS-CoVs and MERS-CoV, hCoV-OC43 can downregulate the transcription of genes critical for the activation of different antiviral signaling pathways [114], and the intragenomic rearrangements described in the intergenic region preceding hCov-OC43 *ns5a* may modulate immune evasion. To this end, hCoV-OC43 ns5a, as well as ns2a, M, or N proteins significantly reduced the transcriptional activity of ISRE, IFN-β promoter, and NF-κB-RE following challenge of human embryonic kidney 293 (HEK-293) cells with Sendai virus, IFN-α or tumor necrosis factor-α [115].

In hCoV-HKU-1 and hCoV-OC43, intragenomic rearrangements involved the intergenic region at the end of the *S* gene highlighting a potential source of regulatory sequences that may affect expression of adjoining genes. The Spike (*S*) gene encodes a structural protein that binds to the host receptors and determines cell tropism as well as the host range. The neighborhood of the spike gene, particularly the region before the S gene, is a hotspot for modular intertypic homologous and non-homologous recombination in coronavirus genomes [34].

Although the nsp3 protein sequence is well conserved among bat *Nobecoviruses*, the significance of the nsp3 segment encoded by the 5′-UTR sequence, which might affect double vesicle membrane formation, remains to be determined. Nsp3 protein, the largest protein encoded by CoVs encompasses up to 16 modular domains. The N-terminal cytosolic domains include a mono-ADP-ribosylhydrolase, a papain-like protease [116], and a scaffold region that participates in replication-transcription complex assembly [117]. After the latter domains, there are two transmembrane domains (TM1 and TM2) with an endoplasmic reticulum luminal loop (Ecto3) between them, and two cytosolic domains (Y1 and CoV-Y) following TM2. The predicted nsp3 segment encoded by the 5′-UTR sequence falls in the cytosolic domain Y1. Nsp3C anchors nsp3 to the endoplasmic reticulum membrane and induces membrane rearrangement leading to double membrane vesicle formation via a yet unknown molecular mechanism [118, 119]. Although there are structural data on the CoV-Y domain [120], its function is unknown as is that of the Y1 domain.

The discontinuous RNA synthesis of the polymerase machinery of coronaviruses along with the use of canonical and noncanonical TRS-L and TRS-B pairing may enhance the occurrence of insertions (via intragenomic rearrangements or other means) and deletions, which can remain uncorrected by the proofreading activity of nsp14 exoribonuclease [121]. Most insertion and deletions likely negatively affect viral fitness [122] and duplication of TRS sequences in coronaviruses led to attenuation [123] and when affecting essential genes frequently to viral genetic instability [124]. However, a small number of insertions/deletions emerge and spread in viral populations, suggesting a positive effect on fitness and adaptive evolution [125–131]. Thus, analyzing these insertion/deletions may reveal evolutionary trends and provide new insight into the surprising variability and rapidly spreading capability that SARS-CoV-2 has shown since its emergence. One usual target of deletions is the accessory ORFs in the distal third of the genome, because they do not appear to participate in viral replication but can allow the virus to evade host defenses. Variants with these deletions occur naturally in SARS-CoV-2 and spread without apparently affecting virus infectivity.

Some of the intragenomic rearrangements described here in *ORF8* and *ORF7a* and one previously in *ORF6* occurred in viruses with deletions that removed or truncated ORFs, such as the deletion in the B.1.36.27 lineage from Hong Kong which lacks *ORFs 7a*, *7b*, and *8* and has the last 12 nucleotides of the *ORF6* replaced by ~ 60 nucleotides from the 5′-UTR [39]. An 872-nucleotide deletion described in the AY.4 lineage (Delta variant) from Southern Poland also eliminated *ORFs 7a*, *7b* and *8* [132], as did a 872-nucleotide deletion documented in late 2021 in Uruguay in a different Delta lineage (AY.20), with viruses without the deletion coexisting with wild-type AY.20 and AY.43 strains [128, 129].

Two large and phylogenetically unrelated deletions (392 and 227 nucleotides long) fused *ORF7a* with downstream ORFs [133]. One, a 392-nucleotide deletion, lacked *ORF7b* and created a new ORF including *ORF7a* and *ORF8*, while the other, a 227-nucleotide deletion, resulted in a new ORF by combining the proximal end of *ORF7a* with *ORF7b*. These deletions have become extinct or appear as sporadic or unique variants [39, 133]. On the other hand, a 382-nucleotide deletion that removes most of the ORF8 was a circulating form hypothesized to lead to an attenuated phenotype of SARS-CoV-2 [130, 131].

Intragenomic rearrangements in isolates with large deletions, as exemplified by those involving *ORF6* [39], *ORF7b* and *ORF8* of SARS-CoV-2, in all cases thus far affect the carboxyl-termini of the predicted encoded proteins. The length of the insertions does not notably affect that of the predicted proteins in isolates without major genomic deletions. For 5′-UTR segments within viral genes, such as the examples shown in *N*, *nsp12* and *nsp3*, or intergenic regions, the length of the protein or intergenic region appears not to be affected.

Intragenomic rearrangements are yet another example of the tremendous genomic flexibility of coronaviruses which underlies changes in transmissibility, immune escape and/or virulence documented during the SARS-CoV-2 pandemic.

### Limitations

The intragenomic rearrangements involving 5′-UTR sequences were detected in all subgenera of β-coronaviruses infecting humans (i.e., *Sarbecovirus*, *Embecovirus*, and *Merbecovirus*) and in the *Nobecovirus* but not the *Hibecovirus* subgenera of CoVs infecting bats. There were only 3 *Hibecovirus* genomes in the database, which may account for the lack of detection of internal rearrangements in this subgenus most closely related to *Sarbecoviruses*. In this respect, the most diverse detection of rearrangements in SARS-CoV-2 may reflect the bias generated by the presence in GenBank of SARS-CoV-2 isolates in up to 5 orders of magnitude greater number than any other CoV. However, the relative paucity of α-, γ-, or δ-CoV sequences available also applies to those of β-CoVs other than SARS-CoV-2 for which 5′-UTR rearrangements were found in notable proportions. Moreover, the present analysis included CoVs involved in large outbreaks such as the swine enteric CoVs of the α and δ genera and avian infectious bronchitis virus of the γ genus that have been studied over decades with hundreds of isolates characterized without apparent evidence for intragenomic rearrangements. The apparent absence of internal rearrangements in the latter viruses bodes well for the specificity of the findings described here for 4 of 5 subgenera of β-CoVs and 3 of 12 subgenera of α-CoVs.

Many sequences in the databases have incomplete 5′-UTRs rendering it difficult to comprehensively analyze them and to calculate more reliable proportions of variations. There are also partial genome and protein sequences, and we excluded sequences with undetermined amino acids. Nonetheless, for SARS-CoV-2, the frequency of variants with full-length insertions appears low relative to those with subsegments or other mutations in comparison to the reference strain in the same insertion area. One could posit that for hCoV-OC43 and hCoV-HKU-1, the apparently much higher frequency of intragenomic rearrangements involving 5′-UTR sequences might be driven by characterization of a greater number of isolates during epidemics with rearrangements possibly providing transmissibility, immune evasion and/or virulence advantages.

A limitation of the methods used for detecting these isolates is that they may not be viable, i.e., they may be associated with molecular diagnostic detection of virus but not necessarily culture conversion, or may represent artifacts of sequencing; however, their prevalence with redundancy in various locations and processing laboratories would be consistent with human-to-human transmission. Moreover, Turakhia et al. [134], among others, have pointed out that systematic errors associated with lab-or protocol-specific practices affect some sequences in the repositories, which are predominantly or exclusively from single labs, co-localize with commonly used primer binding sites and are more likely to affect the protein-coding sequences than other similarly recurrent mutations. Although we cannot rule out that such systematic errors as well as wrong short reads alignment may underlie some if not all the rearrangements detected, the possibility is rendered less likely by the geographic and temporal diversity of the isolates with each intragenomic rearrangement (as underscored by the data in the Additional file 1: legends to Figures and Table), their presence in diverse variants of concern, as well as the occurrence of rearrangements in sequences from before the pandemic era and among diverse viruses of two genera and various subgenera in at least three hosts (humans, bats, and rodents). Moreover, it is unlikely that the insertion in the nucleocapsid gene of SARS-CoV-2 which encodes for a common co-mutation of adjacent sites that has been shown experimentally to have functional significance reflects an artifactual event. Finally, when using peptides as query sequences for SARS-CoV-2 we verified that the nucleotide sequences encoding the detected peptides were identical to 5′-UTR sequences. However, we cannot rule out that the sequences detected in intragenomic rearrangements may have arisen from host cell genomes or other sources.

## Conclusions

We here describe intragenomic rearrangements involving 5′-UTR sequences and the coding section of the genome of beta and alphacoronaviruses. Variation driven by internal rearrangements is distinct from the non-homologous recombination events proposed as origins of *Sarbecovirus*/*Hibecovirus*/*Nobecovirus* β-CoV *ORF3a* by gene duplication followed by rapid divergence from M [34, 135] or of SARS-CoV-2 *ORF8* from *ORF7a* [79]. The mechanisms underlying intragenomic rearrangements warrant further study. Understanding the variation that they introduce also is of relevance in the design of prophylactic and therapeutic interventions for all coronaviruses, including a pan-betacoronavirus vaccine.

## Supplementary Information

**Additional file 1**. Supplementary figures and tables.

## Data Availability

All data generated or analyzed during this study are included in this published article and its Additional file.

## Abbreviations

CoV: Coronavirus
MERS: Middle east respiratory syndrome
N: Nucleocapsid
NIRAN: *Nidovirus* RNA-dependent RNA polymerase associated nucleotidyl transferase
Nts: Nucleotides
ORF: Open reading frame
SARS-CoV-2: Severe acute respiratory syndrome-coronavirus-2
SL: Stem-loop
SR: Serine-arginine
TRS: Transcription regulatory sequence
U/A: Uridine/Adenosine
UTR: Untranslated region
