# Supplementary material for: Intragenomic rearrangements involving 5′-untranslated region segments in SARS-CoV-2, other betacoronaviruses, and alphacoronaviruses
Source: Virol J. 2023 Feb 25;20:36. doi: 10.1186/s12985-023-01998-0 (PMC9957694; doi:10.1186/s12985-023-01998-0)
Supplement: Supplementary file 1 — Additional file 1. Supplementary figures and tables. [file 12985_2023_1998_MOESM1_ESM.docx]

**Supplementary data**

**Supplementary Figure 1**

**A.**

**B.**


**Legend to Supplementary Figure 1**

**A. Discontinuous synthesis of SARS-CoV-2 negative strand subgenomic RNA**. For the synthesis of subgenomic RNA, the leader transcription regulatory sequence (TRS-L, blue box) within the 5’-leader sequence interacts with homologous TRSs in the body (TRS-B) of the genome that precede structural (red boxes) and accessory (green boxes) genes. Overlapping genes for *ORF3a* (namely, *ORF3b-d*) and *N* (namely, *ORF9b, c*) that would be translated from the sgRNAs shown for *ORF3a* and *N*, respectively, are shown at the bottom. **B. Secondary structure of the 5’-UTR including the 5’-leader sequence and translated sequence of the 5’-leader sequence and beyond until the stop codon before stem-loop (SL)5.** The secondary structure of the 5’-UTR is shown as presented in Miao et al. [36]. The 5’ leader sequence extends from the cap structure (m^7^G) to the TRS-L and encompasses SL1-3, which have been associated with viral replication and gene expression. An open reading frame spans most of the 5’-UTR (Wuhan reference strain, NC_045512 shown) except for SL5, where ORF1ab starts. The segment of the open reading frame that is translocated wholly or partially in SARS-CoV-2 variants is underlined. At the nucleotide level, the segment includes the TRS-L, and at the amino acid level, the translated 5’-leader sequence and beyond includes a predicted upstream open reading frame (uORF, grey box) which has not been shown to be functional and whose initiation methionine [M] is shown in gray.

**Supplementary Figure 2**

**A.**

**B.**

**Legend to Supplementary Figure 2**

**A. Most common *ORF8* variants (*ORF8x*) with 5’-UTR sequence intragenomic rearrangement.**

Adenosine and uridine residues are highlighted in fuchsia, while TRS-B and cTRS-B sequences are highlighted in yellow. The nucleotide and amino acid sequence of the 5’-UTR-derived insertions are highlighted in green, while the amino acid sequence for the reference ORF8 is highlighted in yellow. Red asterisks denote stop codons. Number and distribution of collection sites for the most common *ORF* variants are: A. 35 isolates (all USA): California (5), Washington (3), Arizona (3), New York (3), Pennsylvania (2), Florida (2), Texas (2), West Virginia (2), Arkansas (1), South Carolina (1), North Carolina (1), Kansas (1), Georgia (1), Massachusetts (1), New Jersey (1), Nevada (1), Alabama (1), Minnesota (1), Illinois (1), Missouri (1). B. 33 isolates (all USA): Michigan (12), Florida (5), Virginia (2), California (2), Texas (2), Alabama (1), Arizona (1), Ohio (1), New Jersey (1), Illinois (1), Minnesota (1), Massachusetts (1), Arkansas (1), Kentucky (1), Pennsylvania (1). **C.** 106 isolates: California (14), Florida (9), Maryland (8), Pennsylvania (8), Colorado (5), Michigan (5), Minnesota (5), Texas (5), Arizona (3), North Carolina (3), South Carolina (3), Illinois (3), New Jersey (3), Virginia (3), New York (3), New Hampshire (2), Massachusetts (2), West Virginia (2), Tennessee (2), Georgia (2), Indiana (2), Alabama (1), Missouri (1), Utah (1), New Mexico (1), Maine (1), Vermont (1), Kansas (1), Wisconsin (1), District of Columbia (1), Kenya (1), Pakistan (1). C. 33 isolates (all US): Minnesota (10), Maryland (3), Tennessee (3), Georgia (2), South Carolina (2), West Virginia (1), Wisconsin (1), Kentucky (1), Arizona (1), Virginia (1), Michigan (1), Idaho (1), Indiana (1), New Mexico (1), California (1), North Carolina (1), Missouri (1), Colorado (1). **B.** **Alternative structure to that shown in Panel A with TRS-B binding to second cTRS-B.** The minimum free energy is similar to that for TRS-B binding to the first cTRS-B.

**Supplementary Figure 3**

**Legend to Supplementary Figure 3**

**Predicted secondary structures and minimum free energy of the RNAs corresponding to the intragenomic rearrangement in *nsp2* of rodent alphacoronaviruses subgenus *Luchacovirus* and adjacent sequences.**

**Supplementary Figure 4**

**Legend to Supplementary Figure 4**

**A. Summary of locations of intragenomic rearrangements identified in SARS-CoV-2.** Nonstructural structural and accessory genes are represented in yellow, red, and green, respectively. **B. Partial overlap between** t**he 5’-UTR nucleotide segment that is translocated to viral genes and a predicted sequence potentially involved in circularization of genome during viral replication.** The conserved complementary sequences (CCSs) in the 5’- and 3’ UTRs potentially involved in circularization of the genome during subgenomic RNA synthesis are shown. The insertion sequences usually include the TRS-L and span approximately half of the 5’ CCS, thus possibly facilitating circularization of the genome from locations closer to the 3’-UTR.

**Supplementary legend to Figure 1 in main text**

**SARS-CoV-2 variants (collection site and date in parentheses) with 5’-UTR sequence sequences modifying the predicted ORF8 carboxyl terminus; those shown in Figure 1 in main text are highlighted**

**a.** NC_045512 (Wuhan reference); QUP34336 (USA/Minnesota, 2021-04-05);  **b.** QVJ62740 (USA/Michigan, 2021-04-13), QUQ07869 (USA/Florida, 2021-04-04), QTM63997 (USA/Alabama, 2020-10-03), QTZ79380 (USA/Michigan, 2021-03-16), UCP53601 (USA/Arizona, 2020-10-29), QUA38826 (USA/Ohio, 2021-03-09), QTY87294 (USA/New Jersey, 2021-03-18), QTX61599 (USA/Michigan, 2021-02-24), QUA76009 (USA/Illinois, 2021-04-05), QUB00435 (USA/California, 2021-04-011), QVX18739 (USA/Minnesota, 2021-04-16), QVX54931 (USA/Virginia, 2021-05-07), QTW56832 (USA/Michigan, 2021-03-28), QTX80560 (USA/Michigan, 2021-03-08), QSV23506 (USA/Massachusetts, 2021-02-20), QTW56196 (USA/Michigan, 2021-03-28), UBZ78873 (USA/Virginia, 2021-08-15), QTM32955 (USA/California, 2021-03-13), QUP09826 (USA/Michigan, 2021-04-01), QVO97167 (USA/Michigan, 2021-03-10), QTY99266 (USA/Michigan, 2021-03-18), QQE72547 (USA/Florida, 2021-11-17), QUD47078 (USA/Michigan, 2021-04-03), UAZ71320 (USA/Michigan, 2021-03-22), UAP73316 (USA/Texas, 2021-08-20), UER42693 (USA/California, 2021-09-25), UBN91115 (USA/Florida, 2021-03-19), UCZ40652 (USA/Texas, 2021-10-03), QVM43844 (USA/Michigan, 2021-05-02), QZQ76855 (USA/Florida, 2021-05-24), QTB05507 (USA/Arkansas, 2021-03-02), QXG11190 (USA/Kentucky, 2021-05-24), QWA62675 (USA/Florida, 2021-05-08), QTZ60073 (USA/Pennsylvania, 2021-01-23); QUD47078 (USA/Michigan, 2021-04-03); **c.** QTZ60073 (USA/Pennsylvania, 2021-01-23),UBR92989 (USA/California, 2021-07-28), QTW56256 (USA/Pennsylvania, 2021-03-28), QTB05507 (USA/Arkansas, 2021-03-02), QTY29701 (USA/New York, 2021-04-02), QTP28777 (USA/South Carolina, 2021-03-15), QTC77450 (USA/Florida, 2021-02-21), UAU97712 (USA/Kansas, 2021-08-10), QUQ11804 (UA/Georgia, 2021-03-31), UFB90828 (USA/Massachusetts, 2021-10-27), QUC77139 (USA/New York, 2021-03-26), QSG76485 (USA/Texas, 2021-02-13), QRY28882 (USA/Washington, 2021-11-14), QUV66274 (USA/Washington, 2021-02-25), QUE36350 (USA/New Jersey, 2021-04-02), QZF56095 (USA/Nevada, 2021-07-18), QTG51220 (USA/Alabama, 2021-03-09), QTC29731 (USA/Washington, 2021-02-26), QUG22146 (USA/Arizona, 2021-02-03), QTM53955 (USA/California, 2021-03-11), UBG95816 (USA/Texas, 2021-05-27), QUA27428 (USA/New York, 2021-02-26), QYN93853 (USA/Minnesota, 2021-03-16), QWO65210 (USA/Illinois, 2021-05-26), QSW55172 (USA/California, 2021-12-18), QZC48693 (USA/California, 2021-01-08), QTZ59919 (USA/North Carolina, 2021-01-24), UEZ74290 (USA/Florida, 2021-06-13), QVU83077 (USA/West Virginia, 2021-05-03), UCP75589 (USA/Arizona, 2021-09-16), QSL79713 (USA/West Virginia, 2021-02-10), QQV24415 (USA/Washington, 2021-11-03), UDB06943 (USA/California, 2021-01-21), QWU51722 (USA/Missouri, 2021-03-31), QTP78406 (USA/Arizona, 2021-03-22; QWA62675 (USA/Florida, 2021-05-08);  **d.** UAP73316 (USA/Texas, 2021-08-20), UER42693 (USA/California, 2020-09-25); UCZ40652 (USA/Texas, 2021-10-03); **e.** QXY14087 (USA/California, 2021-01-13), QUC83315 (USA/Michigan, 2021-03-26), QQZ32498 (USA/California, 2021-01-08), QQA02853 (USA/Maryland, 2020-11-11), QPG83352 (USA/Florida, 2020-05-29), QRV12235 (USA/Maryland, 2021-01-25), UEK15624 (USA/Colorado, 2020-09-17), QQA02817 (USA/Maryland, 2020-11-11), UCP62018 (USA/Arizona, 2020-10-03), UEK18017 (USA/Colorado, 2020-09-22), UEH39369 (USA/California, 2021-09-28), QZJ67737 (USA/Arizona, 2020-08-03), UFK15561 (USA/Minnesota, 2021-11-05), UFB92196 (USA/North Carolina, 2021-10-27), QZP72779 (USA/Alabama, 2021-07-30), QSX87276 (USA/Pennsylvania, 2021-02-25), UEU99280 (USA/Pennsylvania, 2021-11-06), UEL99603 (USA/California, 2021-10-12), UEU89370 (USA/Minnesota, 2021-11-02), QRG28603 (USA/Florida, 2020-09-05), QUF21578 (USA/Texas, 2021-03-27), UEN39474 (USA/Illinois, 2021-10-10), QUA15764 (USA/Texas, 2021-01-10), QUA44810 (USA/Pennsylvania, 2021-03-15), QVU27766 (USA/North Carolina, 2021-05-01), QUA44621 (USA/North Carolina, 2021-03-14), QYX84583 (USA/New Hampshire, 2021-03-10), QZA59779 (USA/Pennsylvania, 2020-12-03), QSG76473 (USA/Massachusetts, 2021-02-11), QUA13851 (USA/Missouri, 2020-12-06), QPG83362 (USA/Florida, 2020-05-29), QYV28981 (USA/New Jersey, 2021-02-26), QSF03724 (USA/Texas, 2021-02-07), QSL82762 (USA/Pennsylvania, 2021-02-13), QSX25919 (USA/South Carolina, 2021-02-03), QNU10627(USA/Florida, 2021-06-02), QTW58176 (USA/Virginia, 2021-03-29), QZC48492 (USA, California, 2021-01-11), QYM26447 (USA/California, 2020-12-23), QUR38225 (USA/New Jersey, 2021-04-12), QTZ61745 (USA/Arkansas, 2021-02-05), UBX59703 (USA/Minnesota, 2021-09-14), UDA69418 (USA/Virginia, 2021-04-07), QSX71636 (USA/South Carolina, 2021-01-22), QTC08790 (USA/South Carolina, 2021-02-24), QUA17514 (USA/West Virginia, 2021-01-25), UEK12552 (USA/Colorado, 2020-09-22), QSM30820 (USA/California, 2020-12-12-16), QTK20565 (USA/Michigan, 2021-03-16), QSN85839 (USA/Florida, 2021-02-10), QQX23234 (USA/Texas, 2020-10-02), QSF07756 (USA/Pennsylvania, 2021-01-29), QTC79814 (USA/Pennsylvania, 2021-02-22), QQV30431 (USA/California, 2021-01-07), UBE04273 (USA/Tennessee, 2021-09-02), QNL24003 (USA/Maryland, 2020-07-12), QUA19121 (USA/Illinois, 2021-01-29), QSF03424 (USA/California, 2021-02-07), QSO01007 (USA/New Jersey, 2021-02-10), QWO77958 (USA/Utah, 2021-01-26), QZM76691 (USA/New Mexico, 2021-07-19), UCR39596 (USA/Florida, 2021-09-10), UAA61445 (USA/Minnesota, 2021-08-14), QUC86568 (USA/New Hampshire, 2021-03-24), QND77013 (USA/Maine, 2020-03-20), QQN91746 (USA/California, 2020-12-26), UFL93464 (USA/Colorado, 2020-06-15), QXY46820 (USA, New York, 2021-03-23), QZC52177 (USA/California, 2020-07-17), QUH70396 (USA/Michigan, 2021-04-05), QYM30783 (USA/California, 2020-12-08), UET28764 (USA/Pennsylvania, 2021-10-23), QKE12265 (USA/Florida, 2020-04-20), QSX90861 (USA/Massachusetts, 2021-01-12), QUE08549 (UA/Minnesota, 2021-03-28), QSO13388 (USA/Kansas, 2021-02-14), QQH16681 (Pakistan, 2020-04-16), QSJ40506 (USA/Georgia, 2021-02-11), QXI74936 (USA/Arizona, 2021-01-04), UDE63363 (USA/West Virginia, 2021-09-20), QTZ59862 (USA/Florida, 2021-01-24), QQP31916 (USA/Maryland, 2020-12-30), UAJ33375 (USA/California, 2021-08-04), QSX88451 (USA/Pennsylvania, 2021-02-26), QTA53245 (USA/Indiana, 2021-02-04), QSS80876 (USA/Texas, 2021-02-22), QUA15569 (USA/Florida, 2021-01-04), QQN92800 (USA/Tennessee, 2020-11-25), UCO66724 (USA/Indiana, 2021-09-15), QRX62333 (USA/Maryland, 2021-01-31), QTS06851 (USA/Wisconsin, 2021-03-21), QTD08121 (USA/Virginia, 2021-02-28), QWE67901 (USA/District of Columbia, 2021-05-15), QQL13208 (USA/Maryland, 2020-12-01), QVU02466 (USA/Michigan, 2021-04-15), UCN24542 (Kenya, 2020-11-17), UCR20446 (USA/Georgia, 2021-09-03), UCC02971 (USA/New York, 2021-08-26), QTP30077 (USA/Maryland, 2020-11-06), QTY30108 (USA/New York, 2021-03-30), QXS96145 (USA/Vermont, 2021-06-26), QQA02925 (USA/Maryland, 2020-11-11), QUA15581 (USA/New York, 2021-01-04), QUB14694 (USA/Michigan, 2021-03-24), QSJ39354 (USA/Florida, 2021-02-10), QWE91986 (USA/California, 2020-08-29), UEH28437 (USA/Colorado, 2020-09-16), UFD42885 (USA/Illinois, 2021-10-30); **f.** QUD12271 (USA/Ohio, 2021-03/27), QKI36860 (China/Guangzhou, 2020-02-21), QWT91543 (USA/New Mexico, 2020-11-10); UBY86923 (USA/Connecticut, 2021-09-08); QSX87276 (USA/Pennsylvania, 2021-02-25); **g.** UER91358 (USA/Maryland, 2021-10-22); QYF68609 (Bahrain, 2021-06-26); QXG22727 (Bahrain, 2021-06-29); QZP72779 (USA/Alabama, 2021-07-30); UEV0558 (USA/New York, 2021-10-24); UEH58452 (USA/North Carolina, 2021-10-01); **h.** UDN20252 (USA/Minnesota, 2021-10-15), UED44100 (USA/West Virginia, 2021-10-19), UFI73000 (USA/Minnesota, 2021-11-10), UBC64522 (USA/Wisconsin, 2021-07-21), UDL67371 (USA/Kentucky, 2021-09-29), QZS17582 (USA/Maryland, 2021-08-05), UDL43890 (USA/Georgia, 2021-09-26), UFM61198 (USA/Arizona, 2021-10-27), QZS20143 (USA/South Carolina, 2021-08-06), UCN28493 (USA/Minnesota, 2021-09-27), UBD47947 (USA/Virginia, 2021-09-05), UDA13813 (USA/Minnesota, 2021-10-04), QTU42716 (USA/Minnesota, 2021-01-12), UEM47659 (USA/Michigan, 2021-09-23), UDI49835 (USA/Tennessee, 2021-09-21), UDE93735 (USA/Idaho, 2021-10-09), UDA13516 (USA/Minnesota, 2021-10-03), UEQ92175 (USA/Indiana, 2021-10-25), QZS14557 (USA/Maryland, 2021-08-06), UDE67664 (USA/New Mexico, 2021-09-20), UFI75910 (USA/Minnesota, 2021-11-11), UAX90256 (USA/California, 2021-08-12), UBD49836 (USA/Tennessee, 2021-08-31), UDK33208 (USA/North Carolina, 2021-09-23), UDE66381 (USA/Missouri, 2021-09-20), QYS45691 (USA/Minnesota, 2021-07-28), UCK68269 (USA/Minnesota, 2021-08-30), QYY74618 (USA/Georgia, 2021-07-28), UBX72566 (USA/Maryland, 2021-09-01), UDE84787 (USA/South Carolina, 2021-09-21), UEU84720 (USA/Minnesota, 2021-10-31), UDK33636 (USA/Tennessee, 2021-09-23), QZT93722 (USA/Colorado, 2021-08-10); **i.** UDG72468 (USA/Tennessee, 2021-09-13), QTS66472 (USA/California, 2021-03-17), QYY75081 (USA/Georgia, 2021-07-28); QZI47484 (USA/California, 2021-08-16); QUA19334 (USA/Louisiana, 2021-02-02); UBY63352 (USA/Arizona, 2021-09-05); **j.** G (at position 53) in valine codon is replaced by U yielding an L: QTC62354 (USA/California, 2021-02-18); QUF36008 (USA/Michigan, 2021-03-31), QVV00321 (USA/Michigan, 2021-04-22); QSN9715 (USA/Pennsylvania, 2021-02-09); **k.** QQA02853 (USA/Maryland, 2021-11-11), QQS74355 (USA/Maryland, 2021-01-06), QQP31844 (USA/Maryland, 2020-12-29), QQA02817 (USA/Maryland, 2020-11-11); QYM26975 (USA/California, 2020-12-21); QTJ72925 (USA/Massachusetts, 2021-03-10); **l.** QUA15500 (USA/Kansas, 2021-01-02), QTM64537; QVM25420 (USA/Washington, 2021-02-10); QVM48721 (USA/Washington, 2021-03-17).

**Supplementary legend to Figure 3 in main text**

**SARS-CoV-2 variants (collection site and date in parentheses) with 5’-UTR sequence sequences modifying the encoded serine-arginine (SR) region of the predicted N protein; those shown in Figure 3 in main text are highlighted**

**a.** QTO33828 (EPI_ISL_1493720, USA/Texas, 2021/03/14; VOC Alpha GRY [B.1.1.7+Q.*] first detected in the UK), QWQ74880 (EPI_ISL_2528376, USA/Tennessee, 2021-06-03; VOC Gamma GR/501Y.V3 (P.1+P.1.*) first detected in Brazil/Japan), EPI_ISL_2252927 (Canada/Ontario, 2021-04; VOC Gamma GR/501Y.V3 (P.1+P.1.*) first detected in Brazil/Japan); **b.** EPI_ISL_3434731 (Brazil/Espirito Santo, 2021-07-18; VOC Gamma GR/501Y.V3 (P.1+P.1.*) first detected in Brazil/Japan), EPI_ISL_4104739 (Brazil/Sao Paulo, 2021-04-11; VOC Gamma GR/501Y.V3 (P.1+P.1.*) first detected in Brazil/Japan), EPI_ISL_2466134, Brazil/Rio Grande do Sul, 2021-04-15; VOC Gamma GR/501Y.V3 (P.1+P.1.*) first detected in Brazil/Japan), EPI_ISL_4276898 (USA/North Carolina, 2021-07-29; VOC Alpha GRY (B.1.1.7+Q.*) first detected in the UK), EPI_ISL_6050129 (USA/Florida, 2021-04-27; VOC Alpha GRY (B.1.1.7+Q.*) first detected in the UK), EPI_ISL_6050433 (USA/Florida, 2021-04-27; VOC Alpha GRY (B.1.1.7+Q.*) first detected in the UK), EPI_ISL_5676487 (Brazil/Sao Paulo, 2021-07-11; VOC Gamma GR/501Y.V3 (P.1+P.1.*) first detected in Brazil/Japan), EPI_ISL_3487143 (Canada/British Columbia, 2021-05-20; VOC Gamma GR/501Y.V3 (P.1+P.1.*) first detected in Brazil/Japan), EPI_ISL_5649672 (Brazil/Sao Paulo, 2021-05-23; VOC Gamma GR/501Y.V3 (P.1+P.1.*) first detected in Brazil/Japan), EPI_ISL_2493472 (Brazil/Sao Paulo, 2021-05-16; VOC Gamma GR/501Y.V3 (P.1+P.1.*) first detected in Brazil/Japan), EPI_ISL_5802386 (Brazil/Sao Paulo, 2021-04-11; VOC Gamma GR/501Y.V3 (P.1+P.1.*) first detected in Brazil/Japan), EPI_ISL_5802523, Brazil/Sao Paulo, 2021-04-20; VOC Gamma GR/501Y.V3 (P.1+P.1.*) first detected in Brazil/Japan), EPI_ISL_5669813 (Brazil/Sao Paulo, 2021-07-05; VOC Gamma GR/501Y.V3 (P.1+P.1.*) first detected in Brazil/Japan), EPI_ISL_5663882 (Brazil/Sao Paulo, 2021-06-30; VOC Gamma GR/501Y.V3 (P.1+P.1.*) first detected in Brazil/Japan), EPI_ISL_5651350 (Brazil/Sao Paulo, 2021-07-17; VOC Gamma GR/501Y.V3 (P.1+P.1.*) first detected in Brazil/Japan), EPI_ISL_5649266 (Brazil/Sao Paulo, 2021-06-20; VOC Gamma GR/501Y.V3 (P.1+P.1.*) first detected in Brazil/Japan), EPI_ISL_5647661 (Brazil/Sao Paulo, 2021-06-13; VOC Gamma GR/501Y.V3 (P.1+P.1.*), EPI_ISL_7615326 (Brazil/Tocantis, 2021-08-22; VOC Gamma GR/501Y.V3 (P.1+P.1.*) first detected in Brazil/Japan), EPI_ISL_3987895 (Chile/Ñuble, 2021-08-10; VOC Gamma GR/501Y.V3 (P.1+P.1.*) first detected in Brazil/Japan), EPI_ISL_3987894 (Chile/Ñuble, 2021-08-10; VOC Gamma GR/501Y.V3 (P.1+P.1.*) first detected in Brazil/Japan), EPI_ISL_4417129 (Peru/Lima, 2021-08-11; VOC Gamma GR/501Y.V3 (P.1+P.1.*) first detected in Brazil/Japan), EPI_ISL_4414506 (Brazil/Sao Paulo, 2021-07-02; VOC Gamma GR/501Y.V3 (P.1+P.1.*) first detected in Brazil/Japan), EPI_ISL_4413570 (Brazil/Piaui, 2021-08-10; VOC Gamma GR/501Y.V3 (P.1+P.1.*) first detected in Brazil/Japan), EPI_ISL_3259363 (Brazil/Goias, 2021-07-12; VOC Gamma GR/501Y.V3 (P.1+P.1.*) first detected in Brazil/Japan), EPI_ISL_3049003 (Brazil/Sao Paulo, 2021-06-15; VOC Gamma GR/501Y.V3 (P.1+P.1.*) first detected in Brazil/Japan), EPI_ISL_3832398 (Brazil/Rio de Janeiro, 2021-03-15; VOC Gamma GR/501Y.V3 (P.1+P.1.*) first detected in Brazil/Japan), EPI_ISL_2345318 (Brazil/Sao Paulo, 2021-03-21; VOC Gamma GR/501Y.V3 (P.1+P.1.*) first detected in Brazil/Japan), EPI_ISL_2345318 (Brazil/Sao Paulo, 2021-03-21; VOC Gamma GR/501Y.V3 (P.1+P.1.*) first detected in Brazil/Japan), EPI_ISL_3664170 (Brazil/Tocantins, 2021-05-10; VOC Gamma GR/501Y.V3 (P.1+P.1.*) first detected in Brazil/Japan), EPI_ISL_4347353 (USA/Florida, 2021-05-08; VOC Alpha GRY (B.1.1.7+Q.*) first detected in the UK), EPI_ISL_1916295 (India/Maharashtra, 2021-01-06; B.1.1 (Pango v.3.1.20 2022-02-02)), EPI_ISL_1795118 (Brazil/Sao Paulo, 2021-01-06; VOC Gamma GR/501Y.V3 (P.1+P.1.*) first detected in Brazil/Japan), EPI_ISL_295866 (USA/Michigan, 2021-04-16), EPI_ISL_1320623 (USA/North Carolina, 2021-03-02; B.1.1.519 (Pango v.3.1.20 2022-02-02)).

**Supplementary legend to Figure 4 in main text**

**SARS-CoV-2 variants (collection site and date in parentheses) with 5’-UTR sequences modifying the nucleotide segment encoding the N-terminal NiRAN domain of the RNA-dependent RNA polymerase (*nsp12*); those shown in Figure 4 in main text are highlighted**

**a.** QVL75820 (EPI_ISL_1209225, USA/Washington, 2021-03-28), EPI_ISL_1524008 (USA/Washington, 2021-03-28) **b.** UHP90975 (USA/Wisconsin, 2021-12-13), UCX37945 (USA/Wisconsin, 2021-09-20), UET58811 (USA/North Carolina, 2021-10-22), QSF06151 (USA/California, 2021-02-09), QVJ61220 (USA/Tennessee, 2021-04-13), QRW84488 (USA/Massachusetts, 2021-01-17), UBA27920 (USA/Connecticut, 2021-08-26), QTQ63154 (USA/Oregon, 2021-03-05), QUA74029 (USA/Tennessee, 2021-03-30), UHF32869 (USA, 2021-10-07), QSF02035 (USA/North Carolina, 2021-02-04), UCU50446 (USA, 2021-08-07), UDI24491 (USA/Michigan, 2021-09-16),UCX29694 (USA/Wisconsin, 2021-09-17), QUB30213 (USA/Tennessee, 2021-03-16), QTP86894 (USA/Tennessee, 2021-03-23), UHT32237 (USA/Texas, 2021-12-15), UDN70797 (USA/New Jersey 2021-10-02), QSG76884 (USA/Louisiana, 2021-02-11), UHQ26009 (USA/West Virginia, 2021-1206), QQK89755 (USA/Texas, 2020-06-20), QTY89177 (USA/Tennessee, 2021-03-18), UCU60974 (Kenya, 2020-11-12), QQK89671 (USA/Texas, 2020-06-20), UDQ13548 (USA/Massachusetts, 2021-09-30), UHH55298 (USA/Colorado, 2021-10-07), UFL45530 (USA/Wisconsin, 2021-11-07), QUF14763 (USA/Tennessee, 2021-04-09), UIB16394 (USA/California, 2021-12-23), QSN79388 (USA/Louisiana, 2021-02-10), UEL74880 (USA/New York, 2021-10-30), QTC17106 (USA/Pennsylvania, 2021-02-27), QZU12979 (USA/Maryland, 2021-08-08), QTP87948 (USA/Tennessee, 2021-03-23), QXH28738 (USA/Alabama, 2021-04-16), QTQ45244 (USA/Massachusetts, 2021-03-02), UCK93316 (Mexico, 2021-02-10), UDN75390 (USA/Wisconsin, 2021-10-14), UDI22517 (USA/Michigan, 2021-09-16), UEH77441 (USA/Idaho, 2021-10-06), UEI88362 (USA/New York, 2021-10-22), UFO07215 (China/Guangzhou, 2021-05-26), QYR09303 (USA/California, 2021-07-22), QTG30874 (USA/Pennsylvania, 2021-03-03), UFB48090 (USA/Ohio, 2021-11-01), UEN28867 (USA/North Carolina, 2021-10-10), UCX35525 (USA/Michigan, 2021-09-19), UDI24219 (USA/Michigan, 2021-09-16), UFK35880 (USA/Colorado, 2021-10-27), QVP10190 (USA/Tennessee, 2021-03-09), QQD89619 (USA/Texas, 2020-06-19), UDL68518 (USA/Utah, 2021-09-30), UFS19776 (USA/Colorado, 2021-10-19), UFC62618 (USA/North Carolina, 2021-10-17), UFD31246 (USA/Wisconsin, 2021-11-03), UIT57972 (USA/California, 2021-02-15), UBF49486 (USA/Florida, 2021-08-17), QTS23884 (USA/Pennsylvania, 2021-03-17), UFM23938 (USA/Wisconsin, 21021-11-12), QWT32066 (USA/Florida, 2021-04-13), UCL05955 (Mexico, 2021-02-10), QWT32449 (USA/Florida, 2021-04-20), UHY96156 (USA/Indiana, 2021-12-20), QSO02183 (USA/Georgia, 2021-02-15), UHA91620 (USA/Michigan, 2021-02-27), QQD89259 (USA/Texas, 2020-06-19), UFB17148 (USA/California, 2021-09-18), UBI86079 (USA/Washington, 2021-08-24), UFC48585 (USA/Wisconsin, 2021-10-15), QUB35876 (USA/Tennessee, 201-03-18), UET19478 (USA/California, 2021-10-22), QTY89153 (USA/Tennessee, 2021-03-18), QTZ12792 (USA/Tennessee, 2021-03-25), UDN76025 (USA/Wisconsin, 2021-10-15), QUP24690 (USA/Tennessee, 2021-04-03), QRW75822 (USA/Georgia, 2021-01-19), QZQ18874 (USA/Maryland, 2021-07-22), UEH80397 (USA/New Jersey, 2021-10-04), QRW35316 (USA/California, 2021-02-08), QTD08611 (USA/Pennsylvania, 2021-03-01), QZN42224 (USA/Massachusetts, 2021-08-19), QTH26806 (USA/Texas, 2021-03-15), UBA30158 (USA/Connecticut, 2021-08-24), UEH42753 (USA/Utah, 2021-09-29), QRW35868 (USA/California, 2021-02-07), UHN79885 (USA/Wisconsin, 2021-12-09), QSN82400 (USA/Utah, 2021-02-09), UEK36591 (USA/California, 2021-08-25), UGO85135 (USA/Michigan, 2021-03-26), QZN59551 (USA/Massachusetts, 2021-08-21), QTP89015 (USA/New Mexico, 2021-03-22), UHU01313 (USA/New Jersey, 2021-12-09), QUB09583 (USA/Tennessee, 2021-03-22), QUA79262 (USA/Indiana, 2021-03-30), QZR27774 (USA/New Jersey, 2021-08-08), QUF39210 (USA/Virginia, 2021-03-30), QTK00317 (USA/Tennessee, 2021-03-09), QQY92455 (USA/Massachusetts, 2020-10-30), QTP82254 (USA/Indiana, 2021-03-18), QUC73664 (USA/New Jersey, 2021-03-26), UDQ12989 (USA/Texas, 2021-09-30), QSN95378 (USA/Massachusetts, 201-02-11), UHV84541 (USA/South Dakota, 2021-12-17), QTN63096 (USA/Tennessee, 2021-03-16), UEN25043 (USA/California, 2021-10-09), UDI27036 (USA/Michigan, 2021-09-17), UAU82483 (USA/Mississippi, 2021-08-23), UCK93340 (Mexico, 2021-02-12), QSN81011 (USA/Massachusetts, 2021-02-09), QWE98942 (USA/Tennessee, 201-04-01), QUP23047 (USA/Pennsylvania, 2021-04-06), UCK93376 (Mexico, 2021-02-12), QUE01559 (USA/Michigan, 2021-03-25), UIG95057 (USA/Texas, 2020-07-11), UCK93328 (Mexico, 2021-02-12), QSL68204 (USA/Massachusetts, 2021-02-15), UBJ41712 (EPI_ISL_4210459, USA/Colorado, 2021-07-17), UCN32677 (USA/Wisconsin, 2021-09-07), UHB31750 (USA/Wisconsin, 2021-12-06), UGW49737 (USA/Rhode Island, 2021-12-02), QTC69545 (USA/North Carolina, 2021-02-19), QSE23148 (USA/Nevada, 2021-02-02), QTX67843 (USA/Pennsylvania, 2021-02-28), UEO69019 (USA/Vermont, 2021-11-02), QTI92251 (USA/Texas, 2021-03-11), UED49901 (USA/Alabama, 2021-10-11), QUS65424 (USA/Michigan, 2021-04-15), UCL09275 (Mexico, 2021-02-08), UCU26113 (USA/Colorado, 2021-09-15), UBE28012 (USA/Tennessee, 2021-08-31), QZM41370 (USA/Maryland, 2021-08-02), QWP91207 (USA/Utah, 2021-01-12), UCK93017 (Mexico, 2021-02-11), UFL58409 (USA/Georgia, 2021-11-09), UAQ08726 (USA/Kentucky, 2021-08-19), QVQ47721 (USA/Utah, 2021-02-17), UGO30951 (USA/Michigan, 2021-04-09), UCK93268 (Mexico, 2021-02-11), QUC95975 (USA/Wisconsin, 2021-03-29), UCK93256 (Mexico, 2021-02-11), QST20745 (USA/California, 2021-02-16), UFR30090 (USA/Texas, 2021-11-13), UFY60772 (USA/Wisconsin, 2021-11-16), UCK93208 (Mexico, 2021-02-11), UCK93412 (Mexico, 2021-02-09), QYZ51361 (USA/California, 2021-07-22), QSY38005 (USA/Utah, 2021-02-11), QRX36206 (USA/Georgia, 2021-01-26), UCU51547 (USA/California, 2021-08-05), UAJ86375 (USA/Massachusetts, 2021-08-28), UCU60808, UDE70831, UEJ48203, UFG29076, UDN70720, QTS15625, UCV75209, QOL76451, QTP86918, UBI85816 (USA/Washington, 2021-08-25), UHU25439 (USA/Colorado, 2021-12-15), QZH95271 (USA/California, 2021-08-14), UCA83849 (USA/Tennessee, 2021-08-25), UAU14550 (USA/California, 2021-08-07), QTM59778 (USA/California, 2021-03-10), UAB04361 (USA/California, 2021-08-24), QZO45287 (USA/California, 2021-08-16), QZG94711 (USA/California, 2021-08-09), QTM28698 (USA/California, 2021-03-14), UAA69987 (USA/California, 2021-08-20), QZC68055 (USA/California, 2021-08-06), QTM60426 (USA/California, 2021-03-10), QTM35574 (USA/California, 2021-03-19), QZI23057 (USA/California, 2021-08-11), UAC34418 (USA, California, 2021-08-19), QZS28746 (USA/California, 2021-08-11), QZI43852 (USA/California, 2021-08-16), QTM53706 (USA/California, 2021-03-11), QZH97195 (USA/California, 2021-08-14), QTM62250 (USA/California, 2021-03-10), QZS26314 (USA/California, 2021-08-11), QTM46434 (USA/California, 2021-03-09), QZH93082 (USA/California, 2021-08-13), QTM62430 (USA/California, 2021-03-10), QTM28638 (USA/California, 2021-03-14), QTM61734 (USA/California, 2021-03-11), QTM55686 (USA/California, 2021-03-21), QTM51006 (USA/California, 2021-03-03), QZG92437 (USA/California, 2021-08-09), QTQ57981 (USA/California, 2021-03-25), QZC67661 (USA/California, 2021-08-05), UAU22498 (USA/California, 2021-08-09), UAC24682 (USA/California, 2021-08-18), UAU15783 (USA/California, 2021-08-07), QYO40026 (USA/California, 2021-07-26), UAM59368 (USA/California, 2021-08-23), UEW99265 (USA/Minnesota, 2021-09-07), QTM59310 (USA/California, 2021-03-04), QTW87379 (USA/California, 2021-04-05), QTM53802 (USA/California, 2021-03-11), UCM07937 (USA/New Jersey, 2021-09-24), UIA74851 (USA/California, 2021-12-11), QWS07135 (USA/Massachusetts, 2021-05-26), QVO70308 (USA/Massachusetts, 2021-05-10), UIB88512 (USA/California, 2021-12-08), UHD98968 (USA/Washington, 2021-12-10), UHS95021 (USA/California, 2021-12-04), QZI30998 (USA/California, 2021-08-12), QWE74279 (USA/Rhode Island, 2021-05-20), QWT11941 (USA/Rhode Island, 2021-05-19), UHS99543 (USA/California, 2021-12-06), QVU19426 (USA/Tennessee, 2021-05-14), QVK91899 (USA/California, 2021-04-29), UIC02954 (USA/California, 2021-12-11), UDW21718 (USA/Florida, 2021-06-30), UGZ85477 (USA/Washington, 2021-12-02), QVV52552 (USA/Pennsylvania, 2021-05-06), QVU40092 (USA/Tennessee, 2021-05-14), UGV83346 (USA/California, 2021-11-22), QVE27167 (USA/Minnesota, 2021-04-08), UIG78628 (USA/New York, 2021-12-20), UHZ77630 (USA/New York, 2021-12-23), UIO66406 (USA/Colorado, 2021-12-22), UEP72746 (USA/Utah, 2021-10-06), QTQ57299 (USA/California, 2021-03-25), QTW87367 (USA/California, 2021-04-05), QXI81186 (USA/Utah, 2021-01-20), UEZ67112 (USA/Florida, 2021-08-21), QTM59923 (USA/California, 2021-03-10), UAB04274 (USA/California, 2021-08-24), QTM50971 (USA/California, 2021-03-03), QVV13623 (USA/Tennessee, 2021-04-22), UIS90595 (USA/California, 2021-02-23), QSH79120 (USA/Massachusetts, 2020-12-23), QSH79072 (USA/Massachusetts, 2020-12-22), UEO69832 (USA/Vermont, 2021-11-02), QTQ43251 (USA/Massachusetts, 2021-02-16), QTY91588 (USA/Virginia, 2021-03-18), UFD88645 (USA/Florida, 2021-09-01). **c.** UAQ66644 (USA/Virginia, 2021-07-21), QWO72252 (USA/Utah, 2021-01-16), UDN79241 (USA/California, 2021-10-17), UFB02276 (USA/California, 2021-09-28), UBR04551 (EPI_ISL_1700692, USA/California, 2021-04-01), QOT58454 (Australia/Victoria, 2020-08-06), QNO32118 (USA/FL, 2020-07-10), QTF73897 (USA/Washington, 2020-04-17), QOT52503 (Australia/Victoria, 2020-07-29), QOT65712 (Australia/Victoria, 2020-08-04), UAL05318 (USA/New Jersey, 2021-08-04), QVL90312 (USA/Alaska, 2021-02-15), QUG27104 (USA/California, 2021-04-04), QSH79834 (USA/Massachusetts, 2021-02-05), QOT55131 (Australia/Victoria, 2020-08-11), QNP04397 (Australia/Victoria, 2020-07-07), QVJ30557 (USA/Ohio, 2021-04-10), QKV37606 (Australia/Victoria, 2020-03-23), UDN80965 (USA/California, 2021-10-18), UBU58048 (USA/Vermont, 2021-09-24), QTP78529 (USA/Ohio, 2021-03-24), QNO31650 (USA/Florida, 2020-06-03), QIZ13153 (USA/Washington, 2020-03-23), QTP21880 (USA/Pennsylvania, 2021-03-08), QZM66495 (USA/North Carolina, 2021-08-05), UHP32610 (USA/Colorado, 2021-11-26), QOQ08762 (Australia/Victoria, 2020-08-14), QUP30365 (USA/North Carolina, 2021-04-02), UGV53892 (EPI_ISL_7332674, USA/Colorado, 2021-11-18), QKV39238 (USA/Washington, 2020-04-28), QPN00245 (USA/Virginia, 2020-10), QRW69490 (USA/Kansas, 2021-01-02), UFT01738 (EPI_ISL_6911013, USA/Colorado, 2021-11-07), UBN73346 (USA/California, 2021-07-21), QTW98676 (USA/Ohio, 2021-04-08), QZM69710 (USA/North Carolina, 2021-08-07), QOT67656 (Australia/Victoria, 2020-08-11), QTY90842 (USA/Ohio, 2021-03-25), QZJ82123 (USA/Arizona, 2020-11-05), QQW60892 (EPI_ISL_862697, USA/New Mexico, 2021-01-11), QSM36221 (USA/Virginia, 2021-02), QSV97066 (USA/Georgia, 2021-02-25), UHJ22250 (USA/Washington, 2021-11-29), QYB96896 (USA/Nevada, 2021-07-06), QSH74103 (USA/Washington, 2021-02-02), UBG29595 (USA/Pennsylvania, 2021-03-08), UCP53771 (USA/Arizona, 2020-10-29), UFD69116 (USA/Nebraska, 2021-11-01), UET89315 (USA/New Mexico, 2021-10-20), QXL30633 (USA/Nevada, 2021-06-27), QNP05333 (Australia/Victoria, 2020-07-22), UAJ51444 (EPI_ISL_4000736, USA/Texas, 2021-08-07), UIA22551 (USA/Illinois, 2021-12-16), UCA85430 (USA/New Jersey, 2021-08-24), UDA94630 (USA/California, 2021-01-14), QUF14319 (USA/California, 2021-03-30), UEI47887 (USA/Texas, 2021-10-18), UDI32583 (USA/California, 2021-09-23), UEX06825 (USA/Minnesota, 2021-09-15), UEX08512 (USA/Minnesota, 2021-09-15), UDB02101 (Mexico/Baja California, 2020-05-12), UAU26527 (USA/Washington, 2021-07-30), UEF01099 (USA/Vermont, 2021-10-27), UFL76189 (USA/Kentucky, 2021-11-10), UBR04479 (EPI_ISL_1677791, USA/California, 2021-03-29), QTC82985 (USA/Georgia, 2021,02-21), UBS20118 (USA/Washington, 2021-09-06), QUP33452 (USA/Michigan, 2021-04-07), UGY44799 (USA/North Carolina, 2021-08-21), UBR04503 (USA/California, 2021-03-29), QSL69846 (USA/California, 2021-02-08), UCB64272 (USA/North Carolina, 2021-08-25), QTC77717 (USA/North Carolina, 2021-02-21), UCQ05117 (USA/Colorado, 2021-09-13), UBR04539 (EPI_ISL_1700691, USA/California, 2021-0401), QSN97897 (USA/Ohio, 2021-02-04), QUG29808 (USA/Ohio, 2021-04-07), QSX71723 (USA/South Carolina, 2021-01-22), QOT47919 (Australia/Victoria, 2020-08-11), UGW00470 (USA/Vermont, 2021-12-01), UBG86513 (USA/Texas, 2021-06-15), QOT61081 (Australia/Victoria, 2020-08-11), QNP05765 (Australia/Victoria, 2020-07-14), UDO75557 (USA/California, 2021-09-02), QZJ99124 (USA/Arizona, 2021-11-06), QYC02481 (USA/Virginia, 2021-07), QSQ87317 (Spain, 2021-02-06), QSO34191 (USA/Washington, 2021-02-01), UCL05620 (Mexico, 2021-02-10), UDH85977 (USA/Michigan, 2021-10-09), QZM70941 (USA/New York, 2021-08-05), UHQ63083 (USA/Colorado, 2021-11-26), QNP05369 (Australia/Victoria, 2020-07-21), UBT01574 (USA/Vermont, 2021-09-20), QXT01211 (USA/Nevada, 2021-07-03), UGN24690 (USA/Kentucky, 2021-09-08), QZK03982 (USA/Arizona, 2021-11-13), UET58952 (USA/California, 2021-10-22), UEC53187 (USA/California, 2021-10-19), UFN90271 (USA/Vermont, 2021-11-16), UBT66370 (USA/Rhode Island, 2021-09-13), QSX86679 (USA/Connecticut, 2021-02-25), UFY47349 (USA/California, 2021-11-15), QSO06088 (USA/Ohio, 2021-02-10), UEV59764 (USA/Colorado, 2021-10-14), UHR78108 (USA/Arizona, 2021-12-07), QZZ79447 (USA/Minnesota, 2021-08-01), QKV37366 (Australia/Victoria, 2020-03-24), UBC57025 (USA/Oregon, 2021-08-02), QSV31123 (USA/Ohio, 2021-02-03), UFH09963 (USA/California, 2021-11-10), QYK94763 (USA/Arkansas, 2021-07-21), QZR23215 (USA/New Jersey, 2021-08-05), QVJ29898 (USA/California, 2021-04-09), QOQ08846 (Australia/Victoria, 2020-08-15), UFC64772 (USA/Georgia, 2021-10-17), UDK29334 (USA/Oklahoma, 2021-09-22), QNP05189 (Australia/Victoria, 2020-07-22), QRN62752 (USA/Washington, 2020-04-17), QYN93822 (USA/Minnesota, 2021-03-17), UIT15841 (USA/California, 2021-05-07), UFM46861 (USA/Texas, 2021-11-07), UDK29092 (USA/Georgia, 2021-09-22), QUX40238 (USA/Ohio, 2021-04-19), QOT56895 (Australia/Victoria, 2020-08-09), QZK76656 (USA/Arizona, 2021-10-27), UDK82950 (USA/Georgia, 2021-09-20), UFY09470 (USA/Colorado, 2021-11-08), UBR04134 (EPI_ISL_1664598, USA/California, 2021-03-16), QNA41452 (USA/Massachusetts, 2020-05-05), QTG62009 (USA/Minnesota, 2021-03-11), UCI03387 (USA/California, 2021-08-23), UFD69226 (USA/Michigan, 2021-11-01), UHL52806 (USA/Massachusetts, 2021-12-11), QZU53928 (USA/Mississippi, 2021-08-02), UFH62441 (USA/Washington, 2021-11-08), QWO67801 (USA/Texas, 2021-05-25), QOT66936 (Australia/Victoria, 2020-08-04), UBG22777 (USA/Minnesota, 2021-08-12), UAZ00628 (USA/Colorado, 2021-08-10), QKV37702 (Australia/Victoria, 2020-03-25), QOT49983 (Australia/Victoria, 2020-08-18), UAK10370 (USA/Massachusetts, 2021-09-01), QRW53158 (USA/unknown, 2021-12-128), UGA98207 (USA/Pennsylvania, 2021-11-22), QNP05621 (Australia/Victoria, 2020-07-23), QYV26986 (USA/Ohio, 2021-02-19), QWQ56523 (Egypt, 2021-05-03), UCO32484 (USA/South Carolina, 2021-09-08), UHY49505 (USA/California, 2021-11-21), QWQ09843 (USA/New Mexico, 2021-03-03), UBE59357 (USA/Nevada, 2021-06-05), QYP28652 (USA/Florida, 201-07-16), UFP72527 (USA/California, 2021-11-02), QOQ01576 (Australia/Victoria, 2020-07-22), UFB68519 (USA/Washington, 2021-11-04), QZQ30539 (USA/Utah, 2021-08-19), UFD04864 (USA/Maryland, 2021-10-20), UFW04312 (USA/District of Columbia, 2021-11-18), UFB50646 (USA/New Hampshire, 2021-11-01), QSN92962 (USA/Ohio, 2021-02-11), QNP04829 (Australia/Victoria, 2020-07-22), UEZ48750 (USA/Georgia, 2021-08-16), UHF44058 (USA/California, 2021-11-22), UAX63475 (USA/Connecticut, 2021-09-06), QWP94410 (USA/California, 2021-04-19), UHE84670 (USA/Connecticut, 2021-11-10), UFO57784 (USA/Colorado, 2021/10/30), QSO06053 (USA/Ohio, 2021-02-09), QTP21832 (USA/Pennsylvania, 2021-03-08), UIW34244 (USA/Idaho, 2021-10-07), UCL68725 (USA/Vermont, 2021-09-29), QNO72753 (Australia/Victoria, 2020-07-14), UFR51422 (USA/Maryland, 2021-11-13), UGP26607 (USA/California, 2021-11-16), UHJ22059 (USA/Pennsylvania, 2021-11-25), UHA50630 (USA/Massachusetts, 2021-12-03), UET95602 (USA/Nevada, 2021-10-23), QNP04469 (Australia/Victoria, 2020-07-11), UCP63823 (USA/Arizona, 2021-07-21), UDH04229 (USA/Utah, 2021-09-06), UID27151 (USA/Colorado, 2021-12-07), QKV37330 (Australia/Victoria, 2020-03-22), QNP04709 (Australia/Victoria, 2020-07-22), QVK75504 (USA/California, 2021-05-05), UAZ99719 (USA/New York, 2021-04-16), QZI50984 (USA/California, 2021-08-14), QYT26085 (USA/New York, 2021-07-27), UFY76065 (USA/California, 2021-11-17), UBZ72278 (USA/Minnesota, 2021-09-24), UAT65018 (USA/Texas, 2021-08-23), UBZ32334 (USA/Colorado, 2021-09-23), QUX63045 (USA/California, 2021-04-29), QTP21676 (USA/Pennsylvania, 2021-03-08), QUX63246 (USA/California, 2021-04-29), QWQ74846 (USA/Tennessee, 2021-06-03), UHE06838 (Kenya, 2021-04-23), QVM69527 (USA/Washington, 2021-04-10), QTP21868 (USA/Philadelphia, 2021-03-08), QUX78895 (USA/Philadelphia, 2021-04-01), QVO98143 (USA/Utah, 2021-03-09), QTP21928 (USA/Philadelphia, 2021-03-08), UHE10712 (USA/Kansas, 2021-11-19), UCS65869 (Kenya, 2021-04-27), QWQ63966 (USA/Massachusetts, 2021-05-28), QVH96002 (USA/Florida, 2021-04-24), QTT38102 (Austria, 2021-03-14), QVV41697 (USA/Texas, 2021-05-09), UAZ99443 (USA/California, 2021-05-06), UBG20578 (USA/California, 2021-07-07), QXR80990 (USA/New Jersey, 2021-03-15), QUO78350 (USA/North Carolina, 2021-04-15), QTW94838 (USA/Philadelphia, 2021-03-15), QUX78967 (USA/Philadelphia, 2021-04-01), QUX78919 (USA/Philadelphia, 2021-04-01), QUX78955 (USA/Philadelphia, 2021-04-01), QVU55471 (USA/Philadelphia, 2021-05-03), QWS10103 (USA/Connecticut, 2021-05-25), QUD29701 (USA/Pennsylvania, 2021-03-30), QTP21892 (USA/Philadelphia, 2021-03-08), UGW59478 (USA/California, 2020-12-22), UEI21061 (USA/California, 2020-10-27), QZK50146 (USA/Arizona, 2020-10-09), UHF53553 (USA/Illinois, 2021-11-22), UEC91489 (USA/Florida, 2021-10-04), UDD68618 (EPI_ISL_5259201, USA/Colorado, 2021-09-22), UBT74402 (EPI_ISL_4574730, USA/Colorado, 2021-08-09), QVV22431 (EPI_ISL_2229936, USA/Florida, 2021-03-05), UFO92620 (USA/Massachusetts, 2021-10-22), UIT75188 (USA/California, 2021-02-11), QUX63141 (USA/California, 2021-04-29), QUX74660 (USA/California, 2021-04-30), UCN77125 (USA/North Carolina, 2021-09-16), UGP27130 (USA/California, 2021-11-16), UBA22712 (USA/Florida, 2021-08-27), QXN03610 (USA/Nevada, 2021-06-24), QQW26461 (USA/Texas, 2020-10-04). **d.** UHS40780 (USA/California, 2021-09-23), UGO44472, UIW49833, UHZ07618, UHV76469; **e.** UFT72204 (USA/Colorado, 2021-10-27), EPI_ISL_1384819 (India/Maharashtra, 2021-02-12), EPI_ISL_1703925 (India/Maharashtra, 2021-02-07); **f.** QZM71485 (USA/New York, 2021-08-05), QTG28282 (USA/Pennsylvania, 2021-03-06), QVJ64469 (USA/California, 2021-04-14), QRX48545 (USA/Arizona, 2021-01-29), QTJ73204 (USA/Pennsylvania, 2021-03-11), QZX55999 (USA/Utah, 2021/01/28), QUH41414 (USA/California, 2021-04-06), UDA96392 (USA/California, 2021-01-19), QUE38893 (USA/California, 2021-03-31), QOW96201 (USA/Virginia, 2020-08), UDA78757 (USA/California, 2020-12-25), UBD95955 (USA/North Carolina, 2021-08-30), QXR83245 (USA/Pennsylvania, 2021-03-28), QTD04155 (USA/California, 2021-02-24), UDA84951 (Mexico/Baja California, 2020-12-28), UGY48417 (USA/North Carolina, 2021-08-31), UIH05096 (USA/Virginia, 2021-12-06), QVJ64457 (USA/California, 2021-04-14), QTM33114 (USA/California, 2021-03-15), QTM33066 (USA/California, 2021-03-15), QTM51534 (USA/California, 2021-03-02), QTM95171 (USA/California, 2021-03-03), QTM61710 (USA/California, 2021-03-11), QTM32886 (USA/California, 2021-03-15), QWS15650 (USA/Illinois, 2021-05-18), QVN36411 (USA/Illinois, 2021-05-09), QYV41469 (USA/New York, 2021-06-25), QVF50260 (USA/Illinois, 2021-04-26), QWB63840 (USA/Illinois, 2021-05-18), QTM38515 (USA/California, 2021-03-04), QXM96995 (USA/Rhode Island, 2021-06-22), QSN92016 (USA/New Hampshire, 2021-02-10); **g.** UBL67135 (USA/Maryland, 2021-09-07), UBL84726 (USA/Maryland, 2021-09-10), UDK37409 (USA/Maryland, 2021-09-21), QWJ83632 (USA/Florida, 2021-05-11); **h.** UBD35057 (USA/Texas, 2021-09-01), UHR18165 (USA/Maine, 2021-12-10), UBT64155 (USA/Massachusetts, 2021-09-20), UHF10135 (USA/California, 2021-11-15), QTJ77540 (USA/California, 2020-04-27), UBA73421 (USA/Maryland, 2021-09-02), QTJ82390 (USA/California, 2020-04-18), UDF93258 (USA/Maryland, 2021-10-04), UCB61890 (USA/Maryland, 2021-08-27), UBN88464 (USA/Maryland, 2021-04-10), QUI07616 (USA/New Jersey, 2021-04-08).

**Supplementary listing of coronaviruses without intragenomic rearrangements**

Using 5’ UTRs from reference isolates (in parentheses) as query sequences, no 5’-UTR insertions were detected in the genome bodies of other CoVs infecting humans including the *Sarbecovirus* β-CoV SARS-CoV-1 (NC_004718) and the human α-CoVs hCoV-229E (NC_002645, MW532103 and KU291448, subgenus *Duvinacovirus* ) and hCoV-NL63 (NC_005831, 686 isolates, subgenus *Setracovirus*). In addition, no insertions were found in: α-CoVs subgenus *Tegacovirus* feline CoV and infectious peritonitis virus (FECV and FIPV; NC_002306), transmissible gastroenteritis virus (TGEV; DQ811788, NC_038861: 151 isolates), Swine enteric coronavirus strain Italy/213306/2009 (NC_028806, 151 isolates), Canine coronavirus strain CCoV/NTU336/F/2008 (GQ477367, 151 isolates); Alphacoronavirus 1 strain 23/03 (KP849472, 151 isolates), Feline coronavirus strain FCoV/NTU156/P/2007 (GQ152141, 151 isolates), Feline coronavirus strain DF-2 (DQ286389, 151 isolates); Canine coronavirus strain (KC175339, 171 isolates), Feline coronavirus strain FCoV C1Je (DQ848678, 151 isolates), Feline coronavirus isolate 27C (KP143507), Feline coronavirus UU21 HQ012369), Feline coronavirus UU16 (FJ938058), Feline coronavirus UU24 (HQ012370), Feline coronavirus isolate Black (EU186072), subgenus *Rhinacovirus* severe acuate diarrhea syndrome CoV (MK651076), subgenus *Pedacovirus* porcine epidemic diarrhea virus (MK841495), subgenus *Soravirus* Shrew coronavirus isolate Shrew-CoV/Tibet2014 (NC_046955, 1 isolate); subgenus *Sunacovirus* Wencheng Sm shrew coronavirus isolate Xingguo-74 (NC_048211, 10 isolates); subgenus *Luchacovirus* Rodent coronavirus isolate RtMruf-CoV-1/JL2014 (KY370045, 15 isolates), Lucheng Rn rat coronavirus isolate Lijiang-71 (MT820627, 13 isolates), Lucheng Rn rat coronavirus isolate Lucheng-19 (NC_032730, 14 isolates), subgenus *Minacovirus* Mink coronavirus strain WD1127 (NC_023760, 552 isolates), Ferret coronavirus isolate FRCoV-NL-2010 (NC_030292, 10 isolates), Ferret coronavirus (LC215871, 9 isolates), subgenus *Robacovirus* BtRf-AlphaCoV/YN2012 (NC_0268824, 73 isolates), *Rhinolophus affinis* bat CoV HKU2-related isolate 160660 (MN611522, 75 isolates), Porcine enteric alphacoronavirus strain PEAV-GD-CH/2017 (MG742313, 347 isolates), *Rhinolophus* bat coronavirus HKU2 (NC_009988); subgenus *Myotacovirus* Bat alphacoronavirus isolate AMA_L_F (MT862548, 213 isolates), BtMr-AlphaCoV/SAX2011 (NC_028811, 789 isolates); subgenus *Setracovirus* NL63-related bat coronavirus strain BtKYNL63-15 ((KY073746, 266 isolates), subgenus *Colacovirus* Bat coronavirus CDPHE15/USA/2006 (NC_022103, 10 isolates); subgenus *Decacovirus* BtRf-AlphaCoV/HuB2013 (NC_028814, 33 isolates), *Rousettus* bat coronavirus HKU10 (NC_018871, 951 isolates), *Hipposideros* bat coronavirus HKU10 isolate TLC1343A (JQ989272, 937 isolates), *Hipposideros pomona* bat coronavirus HKU10-related isolate 160942 (MN611523, 947 isolates); subgenus *Minunacovirus* Miniopterus bat coronavirus HKU8 (NC_010438, 579 isolates), Bat coronavirus 1B strain AFCD307 (EU420137, 778 isolates); subgenus *Nyctacovirus* Alphacoronavirus Bat-CoV/P.kuhlii/Italy/206645-41/2011 (MH938448, 644 isolates), Alphacoronavirus sp. isolate WA2028 (MK472068, 615 isolates); β-CoVs subgenus *Embecovirus* murine hepatitis virus (MHV; NC_048217; AF208067), rat CoV Parker (NC_006213), rabbit CoV (JN874562), and bovine CoV (BCoV, U00735 and NC_003045; in this case except for sequences in related CoVs like hCoV-OC43 in Figure 8), and subgenus *Hibecovirus* Bat-Hp-betacoronavirus/ZHeijang 2013 (KF636752 and NC_025217) and Zaria bat CoV strain ZBCoV (HQ166910); δ-CoVs subgenus *Buldecovirus* Porcine deltacoronavirus (USA/Ohio444/2014, KR265862; MN942260); Common-moorhen CoV HKU21 (NC_016996); Night-heron CoV HKU19 (NC_016994); Munia CoV HKU13-3514 (NC_011550); Bulbul CoV HKU11-934 ([NC_011547](https://www.ebi.ac.uk/ena/data/view/NC_011547.1)); White-eye CoV HKU16 (NC_016991); Wigeon CoV HKU20 (NC_016995); Sparrow CoV HKU17 strain HKU17-6124 (JQ065045); Sparrow deltacoronavirus strain ISU73347 (MG812378, 176 isolates); and Thrush CoV HKU12-600 (FJ376621), subgenus *Andecovirus* Wigeon coronavirus HKU20 (NC_0169955, 4 isolates), subgenus *Herdecovirus* Night-heron coronavirus HKU19 (NC_016994, 1 isolate); and γ-CoVs subgenus *Igavirus* Infectious avian bronchitis virus (IABV; NC_001452; AY319651) and Turkey CoV (NC_010800), subgenus *Cegacovirus* Beluga Whale CoV SW1 (NC_010646) and Bottlenose dolphin CoV HKU22 isolate CF090327 (KF793825), and subgenus *Brangacovirus* Canada goose coronavirus strain Cambridge_Bay_2017 (NC_046965, 552 isolates).

**Supplementary Table. Number of individual intragenomic rearrangements shown in Figures 1, 3, and 4 and listed in their respective Supplementary legends.**
